# The *Oncidium* Ethylene Synthesis Gene *Oncidium 1-Aminocyclopropane-1 Carboxylic Acid Synthase 12* and Ethylene Receptor Gene *Oncidium ETR1* Affect GA–DELLA and Jasmonic Acid Signaling in Regulating Flowering Time, Anther Dehiscence, and Flower Senescence in *Arabidopsis*

**DOI:** 10.3389/fpls.2022.785441

**Published:** 2022-04-01

**Authors:** Tzu-Hsiang Huang, Wei-Han Hsu, Wan-Ting Mao, Chang-Hsien Yang

**Affiliations:** ^1^Institute of Biotechnology, National Chung Hsing University, Taichung, Taiwan; ^2^Advanced Plant Biotechnology Center, National Chung Hsing University, Taichung, Taiwan

**Keywords:** anther dehiscence, ACC synthase, ethylene response 1, ethylene signaling, *Oncidium* orchids, senescence

## Abstract

In plants, the key enzyme in ethylene biosynthesis is 1-aminocyclopropane-1 carboxylic acid (ACC) synthase (ACS), which catalyzes *S*-adenosyl-L-methionine (SAM) to ACC, the precursor of ethylene. Ethylene binds to its receptors, such as ethylene response 1 (ETR1), to switch on ethylene signal transduction. To understand the function of *ACS* and *ETR1* in orchids, *Oncidium ACC synthase 12* (*OnACS12*) and *Oncidium ETR1* (*OnETR1*) from *Oncidium* Gower Ramsey were functionally analyzed in *Arabidopsis*. 35S::*OnACS12* caused late flowering and anther indehiscence phenotypes due to its effect on GA–DELLA signaling pathways. 35S::*OnACS12* repressed GA biosynthesis genes (*CPS*, *KS*, and *GA3ox1*), which caused the upregulation of DELLA [*GA-INSENSITIVE* (*GAI*), *RGA-LIKE1* (*RGL1*), and *RGL2*] expression. The increase in DELLAs not only suppressed *LEAFY* (*LFY*) expression and caused late flowering but also repressed the jasmonic acid (JA) biosynthesis gene *DAD1* and caused anther indehiscence by downregulating the endothecium-thickening-related genes *MYB26*, *NST1*, and *NST2*. The ectopic expression of an *OnETR1* dominant-negative mutation (*OnETR1-C65Y*) caused both ethylene and JA insensitivity in *Arabidopsis*. 35S::*OnETR1-C65Y* delayed flower/leaf senescence by suppressing downstream genes in ethylene signaling, including *EDF1-4* and *ERF1*, and in JA signaling, including *MYC2* and *WRKY33*. JA signaling repression also resulted in indehiscent anthers *via* the downregulation of *MYB26*, *NST1*, *NST2*, and *MYB85*. These results not only provide new insight into the functions of *ACS* and *ETR1* orthologs but also uncover their functional interactions with other hormone signaling pathways, such as GA–DELLA and JA, in plants.

## Introduction

The gaseous phytohormone ethylene affects many plant developmental processes, including seed germination, seed dormancy, root nodulation, flowering time, fruit ripening, flower/leaf senescence, biotic/abiotic stress responses, and sex determination (Bleecker and Kende, 2000; Guo and Ecker, 2004; Young et al., 2004; Achard et al., 2006, 2007; Corbineau et al., 2014; Li et al., 2019; Feng et al., 2020; Perata, 2020; Chen et al., 2021; Dar et al., 2021).

There are two main steps in the ethylene biosynthetic pathway. The first step is the conversion of *S*-adenosyl-L-methionine (SAM) to 1-aminocyclopropane-1 carboxylic acid (ACC), the precursor of ethylene, by ACC synthase (ACS), and the second step is the further conversion of ACC to ethylene by ACC oxidase (ACO) (Adams and Yang, 1979; Hamilton et al., 1991; Van Der Straeten et al., 1993; Pattyn et al., 2021). In addition to functioning in the ethylene pathway, ACC has been reported to function independently of ethylene as a signaling molecule to regulate several developmental processes, such as pollen tube attraction, guard mother cell terminal division, and early vegetative development (Vanderstraeten et al., 2019; Yin et al., 2019; Mou et al., 2020). ACS has been thought to be a rate-limiting enzyme in ethylene biosynthesis (Adams and Yang, 1979). In plants, the biochemical characterization of ACS and ACO indicates that both are encoded by a divergent gene family (Alexander and Grierson, 2002; Tsuchisaka et al., 2009; Booker and DeLong, 2015; Liu et al., 2015; Houben and Van de Poel, 2019) and are differentially regulated in a tissue-specific manner at the transcriptional, posttranscriptional, and posttranslational levels (Woeste et al., 1999; Yamagami et al., 2003; Rodrigues et al., 2014; Datta et al., 2015; Park et al., 2018; Pattyn et al., 2021). It has been reported that a mutation in maize *ACS* (*ZmACS6*) alters ethylene synthesis and results in delayed leaf senescence (Young et al., 2004). In tomato, an *acs2-1* mutant showed an increased ACS activity and ethylene overproduction, thereby promoting leaf senescence and accelerating fruit ripening (Sharma et al., 2021). In contrast, decreased ACS activity and ethylene production were observed in an *acs2-2* mutant, which showed delayed leaf senescence and fruit ripening (Sharma et al., 2021).

Ethylene triggers ethylene responses by first binding to the endoplasmic reticulum-localized receptors ethylene response 1 (ETR1), ethylene response sensor 1 (ERS1), ETR2, ERS2, and ethylene insensitive 4 (EIN4) to inhibit these receptors from interacting with and activating the negative ethylene signaling regulator constitutive triple response 1 (CTR1; Bleecker, 1999; Chang and Shockey, 1999; Alonso and Stepanova, 2004; Merchante et al., 2013; Ju and Chang, 2015; Yang et al., 2015; Azhar et al., 2019; Binder, 2020; Zhao et al., 2020; Liu and Chen, 2021). The inhibition of the interaction for receptors to CTR1 causes the activation of several positive regulators of the ethylene response downstream of *CTR1*, including *EIN2*, *EIN3*, ethylene response DNA-binding factor *1-4* (*EDF1*-*4*), and ethylene response factors (*ERFs*), resulting in downstream ethylene responses. *EIN2* is a membrane protein that encodes an Nramp family protein (Alonso et al., 1999). *EIN3* encodes a nuclear protein and works downstream of *EIN2* (Chao et al., 1997). The ethylene response DNA-binding factor (*EDF*) family and *ERF* are transcription factors involved in ethylene signal transduction (Stepanova and Alonso, 2005; Chen et al., 2021). In the absence of ethylene, these ethylene receptors (ETR1, ERS1, ETR2, ERS2, and EIN4) can activate CTR1 and suppress ethylene signaling (Binder, 2020; Zhao et al., 2020; Liu and Chen, 2021).

In *Arabidopsis*, *etr1* and *ein2* mutants are insensitive to ethylene and cause delayed leaf/flower senescence (Grbic and Bleecker, 1995; Patterson and Bleecker, 2004; Graham et al., 2012). Interestingly, the ectopic expression of a mutant *Arabidopsis ETR1-1* with cysteine 65 substituted with tyrosine (Cys65Tyr) causes the loss of copper cofactor binding activity to its transmembrane domain and prevents ethylene from binding to itself (Schaller and Bleecker, 1995; Wilkinson et al., 1997; Rodriguez et al., 1999), not only causing an ethylene insensitivity phenotype but also delaying flower and leaf senescence in transgenic tomato, petunia (Wilkinson et al., 1997) and tobacco (Yang et al., 2008). In contrast, mutations in the *CTR1* gene result in a constitutive ethylene response, including early senescence and flower abscission (Kieber et al., 1993; Huang et al., 2003).

Ethylene has been thought to perform its functions by interacting with other plant hormones (Yoo et al., 2009; Van de Poel et al., 2015; Zhou et al., 2019). Ethylene can act synergistically with GAs but antagonistically with ABA in regulating seed germination (Arc et al., 2013; Corbineau et al., 2014; Van de Poel et al., 2015). It has been reported that the downstream gene of ethylene signaling *ERF1* could regulate flowering time in *Arabidopsis* through direct suppression of *FLOWERING LOCUS T* (*FT*) expression (Chen et al., 2021). Ethylene can also regulate gibberellin–DELLA signaling pathways in controlling flowering time (Achard et al., 2007; Yoo et al., 2009). In this case, DELLA proteins could delay the timing of the floral transition, possibly through the suppression of *LEAFY* (*LFY*; Wen and Chang, 2002; Harberd, 2003; Achard et al., 2006) and *WRKY75* (Zhang et al., 2018). Crosstalk between ethylene and auxin in the regulation of root development has also been reported (Ruzicka et al., 2007; Swarup et al., 2007; Mao et al., 2016). Since jasmonic acid (JA) can also promote senescence in *Arabidopsis* (He et al., 2002) and guanine-induced plant immunity to pathogens depends on not only ethylene but also JA signaling (Wang et al., 2022), JA it may also engage in crosstalk with ethylene in regulating these processes.

Genes involved in ethylene biosynthesis and signaling have been characterized and reported in representative dicot/monocot species (Yang et al., 2015; Liu and Chen, 2021; Pattyn et al., 2021), as well as in the charophyte alga *Spirogyra pratensis* (Ju et al., 2015). The possible functional diversity of these genes among different plant species has been reported (Kim et al., 2012; Yang et al., 2015; Liu and Chen, 2021) and remains under investigation. Monocot orchids comprise more than 25,000 species in a large plant family, the *Orchidaceae*. Surprisingly, relatively few molecular investigations of genes involved in ethylene biosynthesis and signaling have been reported (Huang et al., 2007; Lerslerwong and Ketsa, 2008; Almasi and Mohamed, 2020). In this study, we isolated and functionally analyzed *Oncidium ACC synthase 12* (*OnACS12*) and *Oncidium ETR1* (*OnETR1*) from *Oncidium* orchids. We ectopically expressed *OnACS12* and a dominant-negative *OnETR1* mutation (*OnETR1-C65Y*) in *Arabidopsis*. We found that 35S::*OnACS12* could regulate the GA–DELLA signaling pathways controlling flowering time by suppressing *LFY* expression and could regulate anther dehiscence by downregulating the JA synthesis gene *DAD1*. Furthermore, 35S::*OnETR1-C65Y* caused a delay in flower/leaf senescence due to insensitivity to ethylene and JA signaling, which also resulted in anther indehiscence. Thus, our study provides new insight into the functions of *ACS* and *ETR1* orthologs, as well as their interactions with other plant hormones, specifically GA and JA.

## Materials and Methods

### Plant Materials and Growth Conditions

Plants of *Oncidium* Gower Ramsey, a hybrid (*Oncidium Goldiana* × *Oncidium Guinea Gold*; Chang et al., 2010) orchid, were grown at day/night temperature of 26/23°C under long-day conditions in the greenhouse of National Chung-Hsing University, Taichung, Taiwan. *Arabidopsis thaliana* ecotype Columbia was used for all experiments. *Arabidopsis etr1-1* mutants in the Columbia background were obtained from *Arabidopsis* Biological Resource Center, Ohio State University, Columbia, OH, United States. Seeds of *Arabidopsis thaliana* were sterilized by 75% ethanol and placed on agar plates medium (1/2 Murashige-Skoog salt, 1% sucrose, 0.5% agar) with selected antibiotics (Murashige and Skoog, 1962) at 4°C for 2 days. The seedlings were moved into growth chambers under long-day conditions (16 h light/8 h dark) at 22°C for 10–14 days before being transplanted into soil. The light intensity of the growth chambers was 150 μE m^−2^s^−1^.

### Cloning of *OnACS12* and *OnETR1* cDNAs From *Oncidium* Gower Ramsey

Total RNA extracted from approximately 0.1 g of flower buds (fb) from *Oncidium* was used for cDNA synthesis. The full-length cDNAs of *OnACS12* and *OnETR1* were amplified by PCR using the specific 5′ and 3′ primers listed in Supplementary Table 2. Full-length cDNA fragments of *OnACS12* and *OnETR1* were cloned into the linker region in the binary vector pEpyon-12K (CHY Lab, Taichung, Taiwan) under control of the cauliflower mosaic virus (CaMV) 35S promoter for further use in plant transformation.

### Construction of the *OnETR1-C65Y* Construct

To generate *OnETR1-C65Y* fragment, two fragments amplified by PCR using the specific 5′ and 3′ primers listed in Supplementary Table 2 were performed. The front fragment (206 bp) of *OnETR1-C65Y* was PCR-amplified from start codon to the point mutation. The end fragment (1717 bp) of *OnETR1-C65Y* was PCR-amplified from the point mutation to the stop codon. The full-length cDNAs of *OnETR1-C65Y* were then PCR-amplified using the specific 5′ and 3′ primers listed in Supplementary Table 1 and were cloned into the linker region in the binary vector pEpyon-22K (CHY Lab, Taichung, Taiwan) under control of the CaMV 35S promoter for further use in plant transformation.

### Plant Transformation and Transgenic Plant Analysis

The generated 35S::*OnACS12*, 35S::*OnETR1*, and 35S::*OnETR1-C65Y* constructs were transformed into the *Agrobacterium tumefaciens* strain GV3101. These *A. tumefaciens* were then infiltrated into *Arabidopsis* plants through the floral dip method as described previously (Clough and Bent, 1998). Transformants were selected in a medium containing 50 μg ml^−1^ kanamycin and were further verified by PCR and real-time PCR (RT-PCR.)

### Real-Time PCR Analysis

Real-time quantitative PCR was performed on Mini Opticon (Bio-Rad Laboratories, Hercules, CA, United States) and SYBR Green Master Mix (KAPA, Biosystem Wilmington, MA, United States). The amplification condition was 95°C for 3 min, followed by 40 cycles of amplification (95°C for 3 s, 60°C for 30 s, and 72°C for 1 s) and melted (65–95°C with readings every 1°C). Sequences for the primers used for real-time quantitative RT-PCR for *Oncidium* and *Arabidopsis* genes are listed in Supplementary Table 2. The housekeeping gene *UBQ10* was used as normalization control for *Arabidopsis*, and *OnUBQ* gene was used as normalization control for *Oncidium*. Data were analyzed using CFX Manager TM Software (version 1.5, Bio-Rad).^1^

### Confocal Laser Scanning Microscopy

Anthers were imaged by an Olympus FV1000 confocal microscope (Olympus FV1000, Tokyo, Japan). The plant cell cellulose was stained with calcofluor white excited by 405 nm helium/neon laser line, and emission was collected at 415 ~ 470 nm. The lignin was stained with auramine O excited by 488 nm helium/neon laser line, and emission was collected at 470 nm.

### Ethylene Measurements

Ethylene was measured from seedlings. Sixty 12-day-old seedlings were collected, and fresh weight was measured. The sample was placed in an airtight 30-ml flask and incubated for 3 h in growth chamber. By using a gas-tight hypodermic syringe, 1 ml of headspace was sampled from each flask and injected into a gas chromatograph. The gas chromatograph is equipped with an alumina-based capillary column and a flame ionization detector. Three replicates were conducted and the average and SD reported.

### Ethylene Responses

For ethylene responses, mature wild-type (WT) and transgenic *Arabidopsis* plants were sealed in plastic chambers and gassed with air or air containing 6 ppm ethylene for 3 days in a 16 h light/8 h dark cycle as described previously (Chen and Bleecker, 1995; Chen et al., 2011, 2015).

### The Triple Response Assay

For triple responses, WT and transgenic *Arabidopsis* seedlings were grown on MS plates in the presence or absence of 6 mM ethylene precursor ACC for 7 days in the dark (4 days in 4°C, 3 days in 22°C) and phenotype analyzed.

### Assay for Dark-, ACC-, and JA-Induced Leaf Senescence

The dark- and hormone-induced leaf senescence assay was modified as described previously (Li et al., 2013). Detached rosette leaves from mature wild-type and transgenic *Arabidopsis* plants were excised and floated in sealed Petri dishes with 10 ml distilled water in the presence of 75 μM ACC or 50 μM MeJA in the dark for 4 days at 23°C.

### Measurements of Chlorophyll Content

For chlorophyll extraction, leaves from mature wild-type and transgenic *Arabidopsis* plants were incubated in 80% acetone (v/v) for 24 h in darkness as described previously (Li et al., 2013). Absorbance was measured at 645 and 657 nm, and chlorophyll content was calculated based on (20.2 *A_645_ + 8.02* A_657_)/g fresh weight.

## Results

### Isolation of *OnACS12* From *Oncidium* Gower Ramsey

The *Oncidium ACC synthase 12* (*OnACS12*) gene was identified through a search in our *Oncidium* EST database using BLAST with published *Arabidopsis* sequences as templates. The full-length *OnACS12* cDNA encodes 508 amino acids (Supplementary Figure 1) that share 55% similarity with *Arabidopsis ACS12* (AT5G51690.1). *Oncidium* OnACS12 had seven known conserved boxes identified in the ACS orthologs (Supplementary Figure 1). In addition, *OnACS12* has three vital characteristics of ACC synthase: substrate AdoMet specificity (Mccarthy et al., 2001), a pyridoxal phosphate (PLP) binding site (Capitani et al., 1999), and a PLP cofactor active site (Supplementary Figure 1). These results suggested that *OnACS12* functions as an active synthase to produce ACC, the precursor of ethylene. A phylogenetic tree was constructed based on an amino acid sequence alignment and showed that *OnACS12* is closely related to *ACS* in monocots (Supplementary Figure 2).

### Detection of the Expression of *OnACS12* During *Oncidium* Gower Ramsey Flower Development

**Figure 1:**
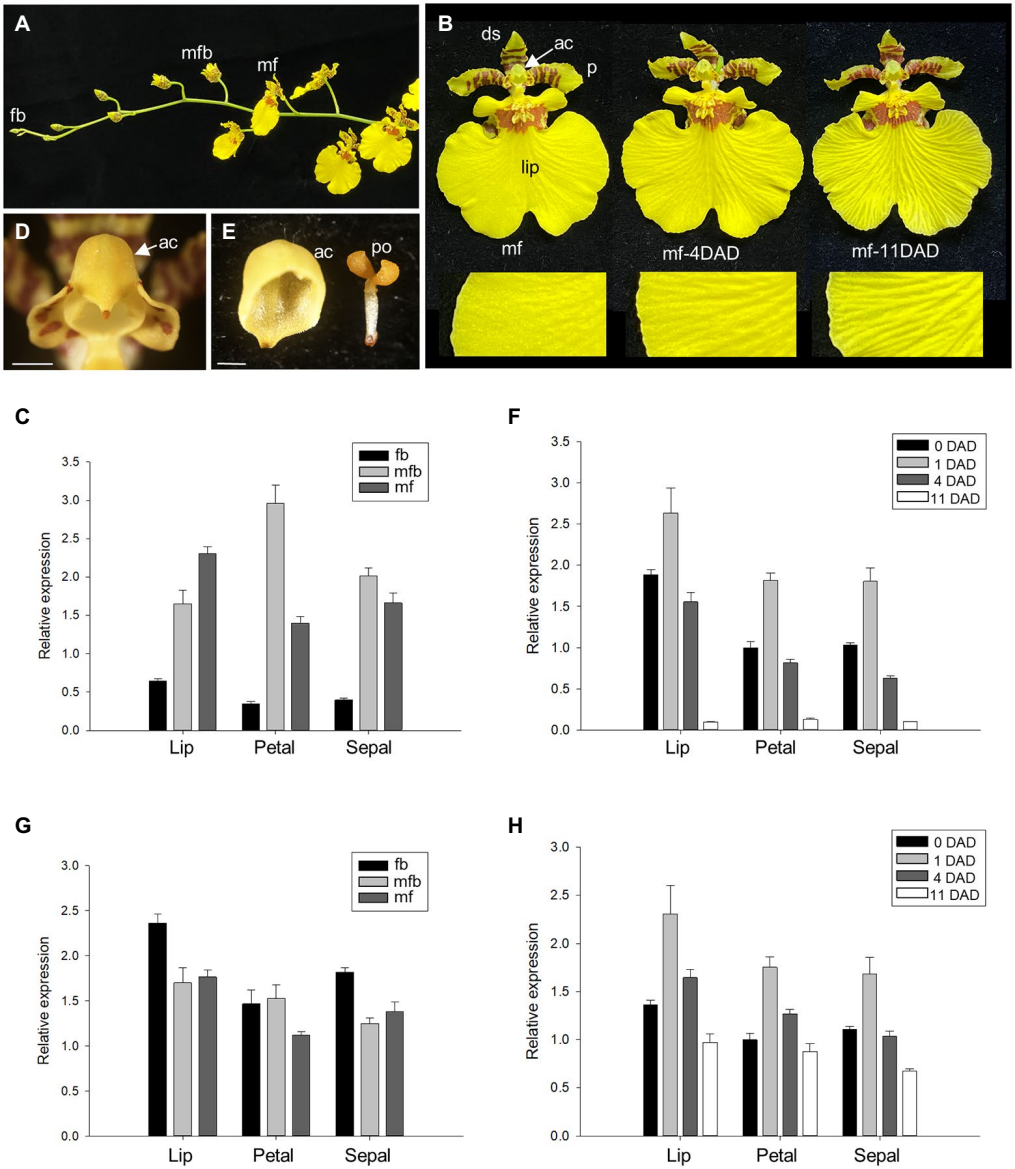
Detection of the expression of *Oncidium ACC synthase 12* (*OnACS12*) and *Oncidium ETR1* (*OnETR1*) during *Oncidium* flower development. **(A)** Flower buds (fb), mature flower buds (mfb), and mature flowers (mf) along an *Oncidium* inflorescence. **(B)** Mature *Oncidium* flower (mf) with the anther cap (ac) attached (left), and 4 days [middle, mf-4 days after anther cap detachment (DAD)] or 11 days (right, mf-11 DAD) after the anther cap detached. A close-up of the lips (lip) is shown at the bottom. p; petals, ds: dorsal sepals. **(C,G)** Detection of the expression of *OnACS12* **(C)** and *OnETR1* **(G)** in the lips, petals, and sepals of *Oncidium* fb, mfb, and mf by real-time quantitative PCR. **(D)** Close up of the anther cap (ac) from (**B**, left). Bar = 0.8 mm. **(E)** Close up of the detached anther cap (ac) and pollinium (po). Bar = 0.6 mm. **(F,H)** Expression of *OnACS12* **(F)** and *OnETR1* **(H)** in the lips, petals, and sepals of flowers 0, 1, 4, and 11 DAD as detected *via* real-time quantitative PCR. In **(C,F,G,H)**, the transcript levels of the genes were determined using two to three replicates and were normalized to the *OnUBQ* gene. The error bars represent SD.

To determine the expression profile of *OnACS12*, *OnACS12* expression was detected in different flower organs (lips/sepals/petals) of *O*. Gower Ramsey flowers at different developmental stages (Figures 1A,B). *OnACS12* was expressed in all flower organs in young fb, mature flower buds (mfb), and mature flowers (mf; Figure 1C). *OnACS12* expression was relatively low in the floral organs of young flower buds and increased in mfb and in mf (Figure 1C). This result indicated that having the lowest expression of *OnACS12* in young flower buds ensured that senescence will not occur during early *Oncidium* flower development.

It has been reported that *Oncidium* and *Cymbidium* flower senescence is induced by the detachment of the anther cap (ac; Figures 1B,D,E; Woltering, 1990; Huang et al., 2007). To further analyze the relationship between flower senescence and the expression levels of *OnACS12*, mRNA from flower organs on different days after ac detachment (DAD; Figure 1B) was used to detect the expression of *OnACS12*. The results showed that *OnACS12* expression was clearly upregulated at one DAD (Figure 1F), at which time no sign of senescence in the perianth was observed. *OnACS12* expression was decreased at 4 DAD (Figure 1F), when slight senescence occurred in the perianth (Figure 1B, middle), and it was significantly downregulated at 11 DAD (Figure 1F), when flower organs were severely senescent (Figure 1B, right). This result indicated that anther cap detachment could induce the expression of *OnACS12* and start the senescence process very quickly. Once the process was initiated, *OnACS12* expression gradually decreased to its lowest level when the flower was severely senescent.

### Ectopic Expression of *OnACS12* Increased Ethylene Production

**Figure 2:**
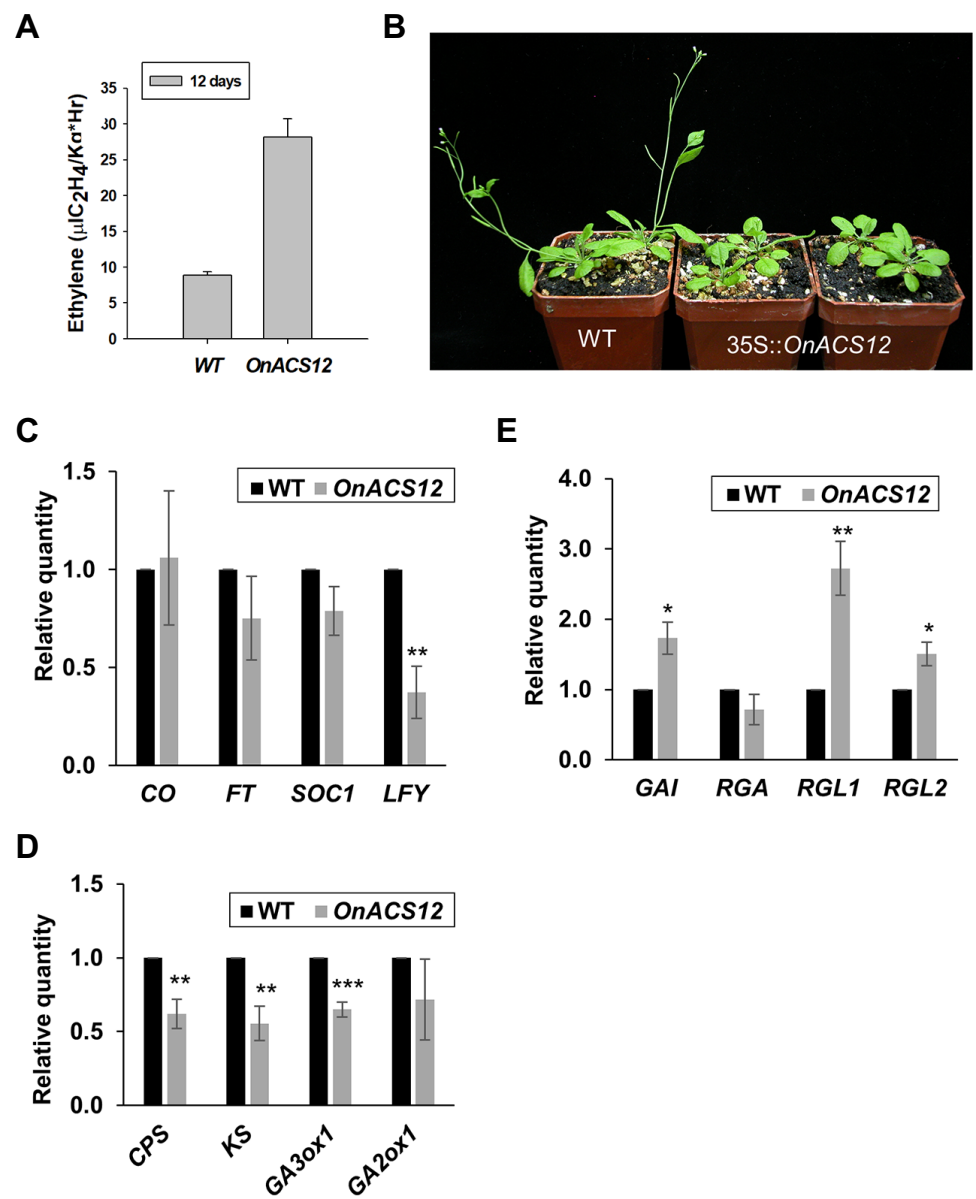
Phenotypic analysis and detection of gene expression in *Arabidopsis* ectopically expressing *OnACS12*. **(A)** Detection of ethylene production in 12-day-old wild-type (WT) and 35S::*OnACS12* seedlings. **(B)** Four 38-day-old 35S::*OnACS12* plants (middle and right) flowered at 43 days, much later than wild-type plants (left) when grown under LD conditions. **(C–E)** Detection of the expression of flowering time genes, GA biosynthesis genes and DELLAs *via* real-time quantitative PCR. Total RNA isolated from 35S::*OnACS12* transgenic *Arabidopsis* and WT Columbia plants was used as a template to detect the expression of the flowering time genes *CONSTANS* (*CO*), *FLOWERING LOCUS T* (*FT*), *SUPPRESSOR OF OVEREXPRESSION OF CONSTANS* (*SOC1*), and *LFY* **(C)**, the GA biosynthetic genes *CPS*, *KS*, *GA3ox1*, and *GA2ox1* **(D)** and the DELLAs *GA-INSENSITIVE* (*GAI*), *REPRESSOR OF GA1-3* (*RGA*), *RGA-LIKE1* (*RGL1*), and *RGL2* **(E)**. The transcript levels of these genes were determined using two to three replicates and were normalized to *UBQ10*. The expression of each gene in the transgenic plants is given relative to that of the wild-type plant, which was set at 1. The error bars represent SD. The asterisks indicate a significant difference from the WT value (^*^p < 0.05; ^**^p < 0.01; and ^***^p < 0.001). Statistical analysis was conducting using two-tailed Student’s *t*-tests.

To further explore its role, *OnACS12* cDNA driven by the CaMV 35S promoter was constructed and transformed into *Arabidopsis*. To verify ethylene synthesis in 35S::*OnACS12 Arabidopsis*, we measured ethylene production from wild-type and 35S::*OnACS12* transgenic *Arabidopsis*. The results indicated that ethylene production was 28.20 μl C_2_H_4_/kg*Hr on average for 35S::*OnACS12 Arabidopsis*, which was much higher than that of wild-type *Arabidopsis* (8.91 μl C_2_H_4_/kg*Hr on average; Figure 2A). This result indicated that the ectopic expression of *OnACS12* could increase ethylene production in *Arabidopsis*.

### Ectopic Expression of *OnACS12* Delayed Flowering Time in *Arabidopsis*

In this study, 80 independent *OnACS12* transgenic *Arabidopsis* T1 plants were obtained. Fifty-four plants were phenotypically indistinguishable from wild-type plants, whereas the other 26 plants showed a late flowering phenotype (Figure 2B). 35S::*OnACS12 Arabidopsis* flowered 39 days after germination on average, which was obviously later than that of wild-type plants, which flowered at 31 days (Supplementary Table 3). To explore the relationship between *OnACS12* and flowering time genes, the expression of *CONSTANS* (*CO*), *FT*, *SUPPRESSOR OF OVEREXPRESSION OF CONSTANS* (*SOC1*), and *LFY* was analyzed using quantitative RT–PCR. As shown in Figure 2C, the expression of *LFY* was significantly downregulated, whereas the expression of *FT* and *SOC1* was slightly suppressed in transgenic 35S::*OnACS12* plants. This result indicated that the late-flowering phenotype of 35S::*OnACS12* was mainly correlated with the downregulation of *LFY*.

### Late Flowering of 35S::*OnACS12 Arabidopsis* Was Due to Alteration of the Gibberellin–DELLA Signaling Pathway

It has been thought that ethylene acts as a plant stress hormone and delays flowering by regulating gibberellin–DELLA signaling (Achard et al., 2007; Yoo et al., 2009). GA has been reported to promote the development and elongation of flower organs by suppressing the functions of DELLA proteins (Cheng et al., 2004; Tyler et al., 2004), which contain an N-terminal DELLA domain responsible for the activity of the DELLA proteins in response to GA (Peng et al., 1997; Silverstone et al., 1998; Dill and Sun, 2001). DELLA proteins act as repressors of GA signaling and delay the timing of the floral transition, possibly through the downregulation of *LFY* (Wen and Chang, 2002; Harberd, 2003; Achard et al., 2006). Since 35S::*OnACS12* increased ethylene production and delayed flowering time, exploring the relationship between *OnACS12* and gibberellin–DELLA signaling pathways was necessary. When the expression of three GA biosynthesis genes, *CPS*, *KS*, *GA3ox1*, and one GA deactivation gene, *GA2ox1*, was analyzed in 35S::*OnACS12 Arabidopsis*, a clear downregulation of *CPS*, *KS*, and *GA3ox1* was observed (Figure 2D). The expression of *GA2ox1* was not affected (Figure 2D). When the expression of the *DELLA* genes *GA-INSENSITIVE* (*GAI*), *REPRESSOR OF GA1-3* (*RGA*), and *RGA-LIKE1* (*RGL1*) and *RGL2* was further analyzed, the expression levels of *GAI*, *RGL1*, and *RGL2* were clearly upregulated in 35S::*OnACS12* plants (Figure 2E). These results implied that *OnACS12* reduced the levels of bioactive GA and promoted *DELLA* gene expression to further suppress *LFY* expression and delay flowering.

### 35S::*OnACS12* Caused Male Sterility Due to the Production of Indehiscent Anthers

**Figure 3:**
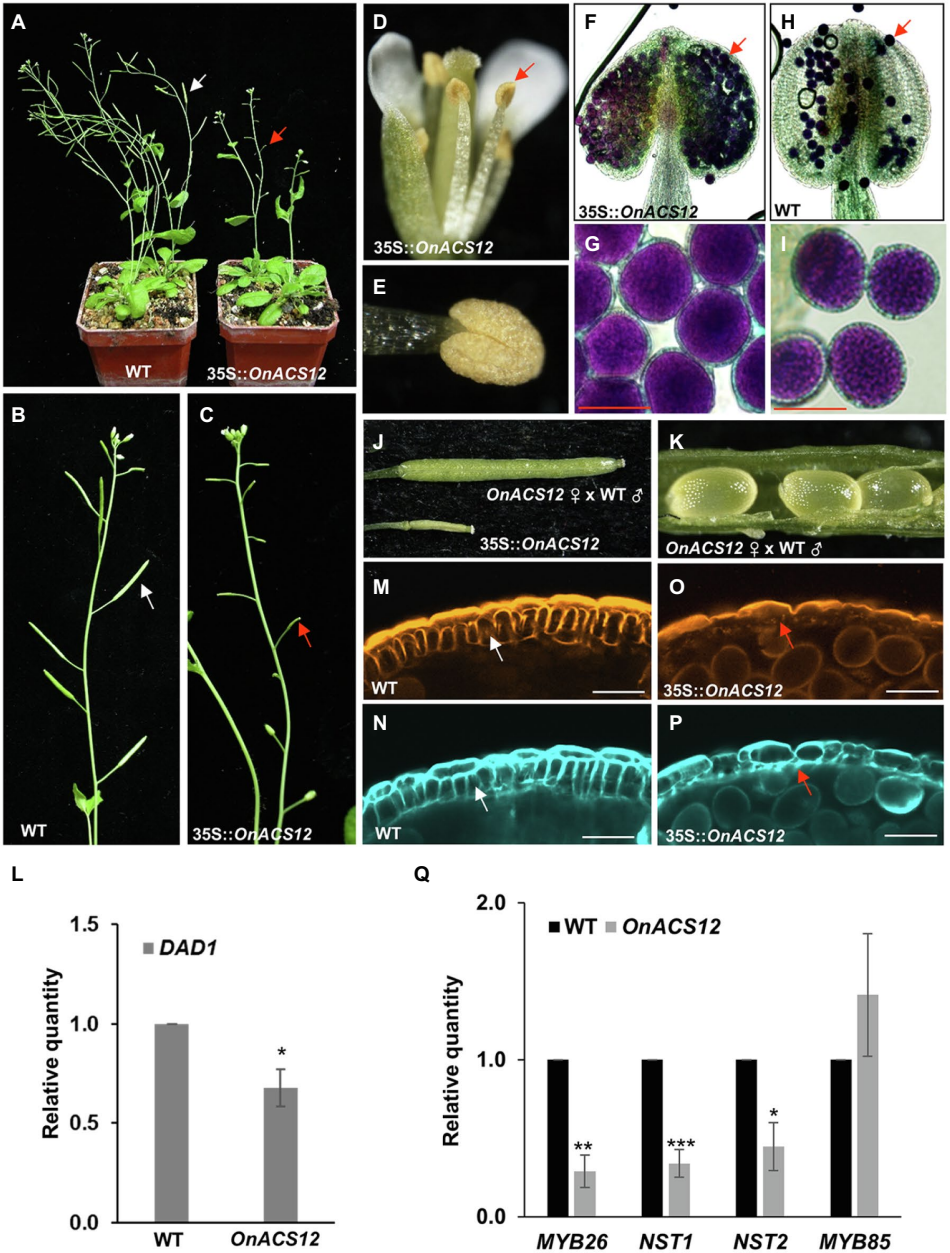
Analysis of the indehiscent anther phenotype and detection of gene expression in 35S::*OnACS12 Arabidopsis*. **(A)** A 52-day-old 35S::*OnACS12* plant produced short siliques (red arrow on the right), whereas the wild-type plant produced normal elongated siliques (white arrow on the left). **(B,C)** Wild-type inflorescence showing elongated siliques (white arrow in **B**) and a 35S::*OnACS12* plant with short siliques (red arrow in **C**). **(D)** A 35S::*OnACS12* flower with an indehiscent anther (arrow). **(E)** Close-up view of an indehiscent anther from **(D)**. **(F–I)** Pollen grain viability analysis of 35S::*OnACS12* male-sterile anthers *via* Alexander staining. Anthers of 35S::*OnACS12* **(F)** and wild-type **(H)** *Arabidopsis* had similar viable pollen grains (arrows). Close-up of pollen grains with similar shapes and normal viability from 35S::*OnACS12* **(G)** and wild-type **(I)** plants. Bar = 20 μm. **(J)** The normal elongated silique of a 35S::*OnACS12* plant after cross-pollination with wild-type pollen (top) along with a short 35S::*OnACS12* silique without pollination (bottom). **(K)** Normal seed development was observed in a 35S::*OnACS12* silique after cross-pollination. **(L,Q)** The mRNA levels were determined *via* real-time quantitative PCR. Total RNA isolated from 35S::*OnACS12* transgenic plants and wild-type Columbia plants was used as a template to detect the expression of *DAD1* **(L)**, *MYB26*, *NST1/2*, and *MYB85* **(Q)**. The transcript levels of these genes were determined using two to three replicates and were normalized to *UBQ10*. The expression of each gene in the transgenic plants is given relative to that of the wild-type plant, which was set at 1. The error bars represent SDs. The asterisks indicate a significant difference from the WT value (^*^p < 0.05; ^**^p < 0.01; and ^***^p < 0.001). Statistical analysis was conducting using two-tailed Student’s *t*-tests. **(M–P)** Confocal laser-scanning microscopy (CLSM) of anther cell walls in 35S::*OnACS12* **(O,P)** and wild-type **(M,N)** plants. The anthers were double stained with auramine O for lignin, indicated in orange **(M,O)**, and calcofluor white for cellulose, indicated in cyan **(N,P)**. Secondary thickening is visible in the endothecium (arrow) of the wild-type anthers **(M,N)** and is absent in 35S::*OnACS12* anthers (arrow; **O,P**). Bar = 25 μm.

In addition to delaying the flowering time, 35S::*OnACS12 Arabidopsis* also showed a sterility phenotype by producing unelongated siliques during late development (Figures 3A,C; Supplementary Figures 3A–D); these siliques were significantly different from the elongated and fully developed siliques of the wild-type plants (Figures 3A,B). 35S::*OnACS12* flowers produced normal sepals, petals, and carpels (Figure 3D). However, the anthers on the stamens were indehiscent at all stages of flower development (Figures 3D,E). Thus, the 35S::*OnACS12* flowers were sterile and unable to set seeds due to the inability to successfully pollinate. Alexander’s staining indicated that the indehiscent anthers of 35S::*OnACS12 Arabidopsis* contained viable pollen grains (Figures 3F,G) that closely resembled wild-type pollen (Figures 3H,I). To further examine pistil activity, wild-type pollen was crossed with stigmas of sterile 35S::*OnACS12 Arabidopsis*. The siliques exhibited normal elongation and produced normal seeds after cross-pollination (Figures 3J,K; Supplementary Figures 3E,F). These results indicated that the sterility of 35S::*OnACS12* plants was associated with indehiscent anthers.

### The JA Biosynthesis Gene *DAD1* Was Downregulated in 35S::*OnACS12* Plants

The anther indehiscence phenotype in 35S::*OnACS12* plants was similar to that observed in mutants of the *Arabidopsis* JA biosynthesis gene *DAD1* (Ishiguro et al., 2001); GA has been reported to be able to regulate anther dehiscence by repressing the DELLA proteins and sequentially activating the JA biosynthesis gene *DAD1* (Cheng et al., 2009; Peng, 2009; Marciniak and Przedniczek, 2019); and GA was suppressed, whereas DELLA genes were upregulated in 35S::*OnACS12* plants. Thus, the relationship between *OnACS12* and *DAD1* expression was analyzed in 35S::*OnACS12* flowers. As shown in Figure 3L, the level of *DAD1* transcripts was clearly downregulated in 35S::*OnACS12* flowers. This result indicated that the anther indehiscence in 35S::*OnACS12* plants was likely due to the suppressed expression of the JA biosynthesis gene *DAD1*, which was caused by the decrease in GA levels and increased DELLA activity in 35S::*OnACS12* plants.

### 35S::*OnACS12* Caused Indehiscent Anthers Due to the Failure of Secondary Cell Wall Thickening Through the Downregulation of *MYB26*, *NST1*, and *NST2* Expression

Anther dehiscence is the final action of the anther and requires the lignification and secondary cell wall thickening of the endothecium to generate forces to rupture the anther cell wall and permit pollen release (Bonner and Dickinson, 1989; Yang et al., 2007; Cecchetti et al., 2013). To further examine the secondary wall thickening and analyze the cellular basis for anther dehiscence, lignin staining with auramine O and cellulose staining with calcofluor white were performed in the endothecium of developing anthers in 35S::*OnACS12* and wild-type plants. The results indicated that in wild-type plants, secondary thickening occurred in the endothecium, with striated lignin formation before anther dehiscence, and the surrounding cell layers of the anther did not undergo secondary thickening (Figures 3M,N). In contrast, the secondary cell wall could not thicken and lignin formation was absent in the endothecium of 35S::*OnACS12* plants (Figures 3O,P).

*MYB26*, *MYB85*, *NAC SECONDARY WALL PROMOTING FACTOR1* (*NST1*), and *NST2* have been reported to be involved in regulating secondary thickness in the anther endothecium (Mitsuda et al., 2005; Zhong et al., 2008). *nst1nst2* double mutants exhibited male sterility, with a similar anther-indehiscent phenotype as that of 35S::*OnACS12* plants, due to the loss of secondary wall thickening in the anther endothecium (Mitsuda et al., 2005). To confirm whether the failure of secondary thickening in the anther endothecium of 35S::*OnACS12* plants was associated with the altered expression of the *MYB26*, *MYB85*, *NST1*, and *NST2* genes, the expression of these genes was analyzed in 35S::*OnACS12* flowers. The results indicated that the expression of *MYB26*, *NST1*, and *NST2* was downregulated in 35S::*OnACS12* plants, whereas the expression of *MYB85* was unaffected (Figure 3Q). Thus, *OnACS12* also caused indehiscent anthers due to the failure of secondary cell wall thickening through the downregulation of *MYB26*, *NST1*, and NST2 in *Arabidopsis*.

### Isolation of *OnETR1* From *Oncidium* Gower Ramsey

The *Oncidium ETR1* (*OnETR1*) gene was identified through a search in our *Oncidium* EST database using BLAST with published *Arabidopsis* sequences as templates. The full-length *OnETR1* cDNA encodes 631 amino acids (Supplementary Figure 4) that share 71% similarity with *Arabidopsis ETR1* (AT1G66340.1). *Oncidium OnETR1* had four conserved domains, including an ethylene binding site, a GAF domain, a histidine kinase domain, and a cyan box, which were identified as putative receiver domains in the ETR1 orthologs (Supplementary Figure 4; Parkinson and Kofoid, 1992). Sequence and domain conservation suggested that *OnETR1* functions as an *ETR1* ortholog in *Oncidium*. A phylogenetic tree was constructed based on an amino acid sequence alignment and showed that *OnETR1* is closely related to the *ETR1* orthologs from the orchids *Dendrobium* (*DeETR*) and *Phalaenopsis* (*PhETR*; Supplementary Figure 5).

### Detection of *OnETR1* Expression During *Oncidium* Gower Ramsey Flower Development

To determine the expression profile of *OnETR1*, *OnETR1* expression was detected in different flower organs at different developmental stages in *O*. Gower Ramsey flowers (Figures 1A,B). In contrast to *OnACS12*, *OnETR1* was expressed in all flower organs (lips/sepals/petals) at a similar level in young flower buds, mature flower buds, and mature flowers (Figure 1G). When the expression level of *OnETR1* from flower organs on different DAD (Figure 1B) was examined, *OnETR1* expression was upregulated in all flower organs at one DAD (Figure 1H), similar to *OnACS12* expression (Figure 1F). This result may due to the effect of a negative feedback loop for ethylene signaling (Azhar et al., 2019). It has been reported that this negative feedback loop could desensitize plants to ethylene and functioned at the level of the ethylene receptors (Azhar et al., 2019). Thus, early after the removing of the anther cap may increase the ethylene level and sequentially cause the upregulation of ethylene receptor *OnETR1* in *Oncidium* through the negative feedback loop mechanism. However, at 4 and 11 DAD, *OnETR1* expression decreased in the flowers to a level similar to that observed in control flowers at 0 DAD (Figure 1H). These results indicated that as a receptor, *OnETR1* was constitutively expressed at a certain level during different stages of *Oncidium* flower development, as well as during the entire process of flower senescence.

### A Dominant-Negatives 35S::*OnETR1-C65Y* Mutation Delayed Flower Senescence by Suppressing Ethylene Signal Transduction in *Arabidopsis*

**Figure 4:**
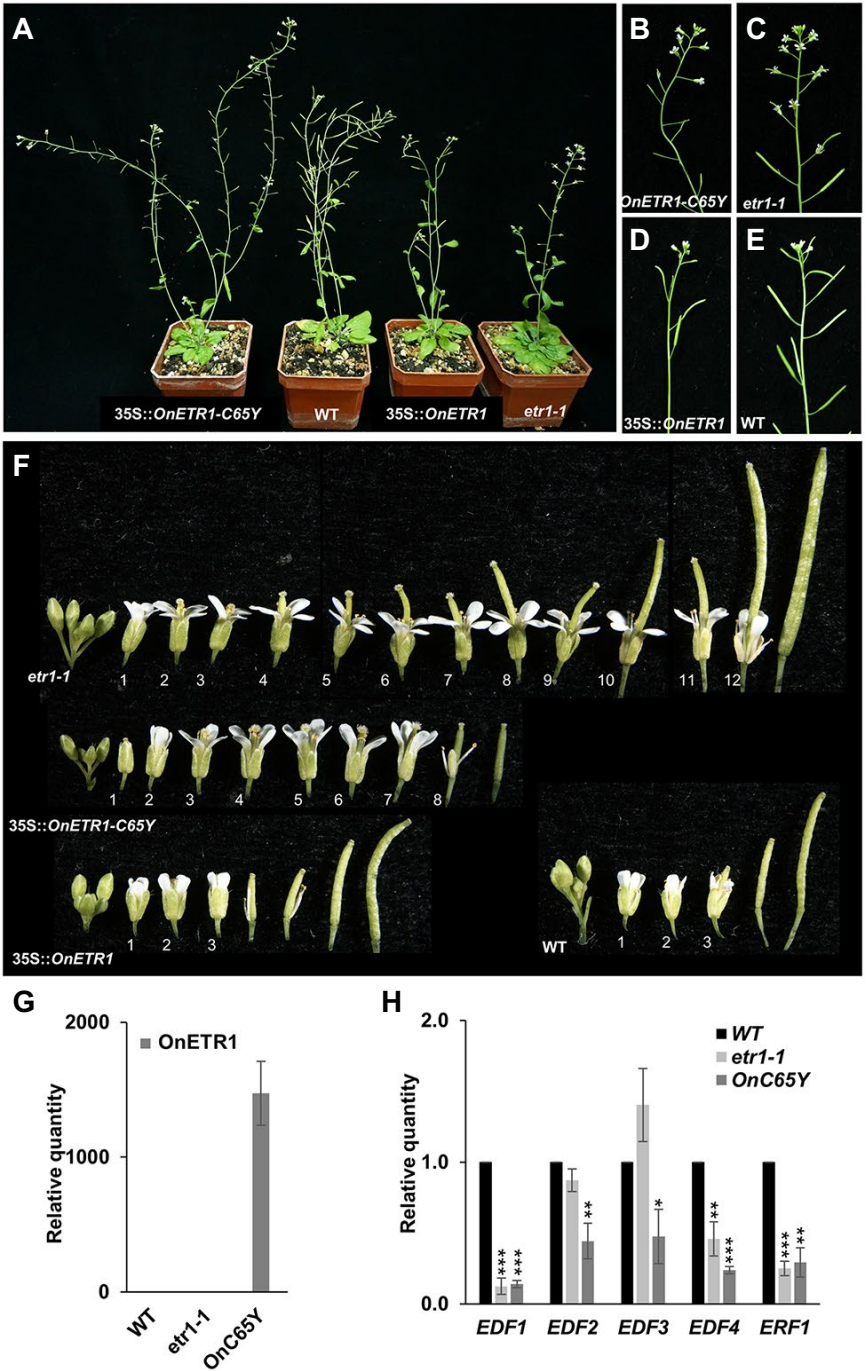
Phenotypic analysis and detection of gene expression in *Arabidopsis* ectopically expressing *OnETR1-C65Y*. **(A)** Mature 35S::*OnETR1*-*C65Y*, WT, 35S::*OnETR1*, and *etr1-1* plants (from left to right). **(B–E)** Close-up of the inflorescences of the 35S::*OnETR1*-*C65Y* **(B)**, *etr1-1* **(C)**, 35S::*OnETR1* **(D)**, and wild-type **(E)** plants from **(A)**. **(F)** Flowers along inflorescences from *etr1-1*, 35S::*OnETR1*-*C65Y*, 35S::*OnETR1* and WT *Arabidopsis*. The senescence and abscission of flower organs in *etr1-1* and 35S::*OnETR1*-*C65Y* flowers were significantly delayed. The numbers indicate the positions of the flowers. **(G)** Relative expression of *OnETR1*-*C65Y* in WT, *etr1-1* and 35S::*OnETR1-C65Y* transgenic *Arabidopsis* based on real-time quantitative PCR. **(H)** Relative expression of the downstream ethylene signaling genes *EDF1-4* and *ERF1* in WT, *etr1-1* and 35S::*OnETR1-C65Y* transgenic *Arabidopsis* based on real-time quantitative PCR. The transcript levels of these genes were determined using two to three replicates and were normalized to *UBQ10*. The expression of each gene in the transgenic plants is given relative to that of the wild-type plant, which was set at 1. The error bars represent SDs. The asterisks indicate a significant difference from the WT value (^*^p < 0.05; ^**^p < 0.01; and ^***^p < 0.001). Statistical analysis was conducting using two-tailed Student’s *t*-tests.

To further explore the role of *Oncidium OnETR1*, a dominant-negative mutation, *OnETR1-C65Y*, in which cysteine 65 was substituted with tyrosine, was generated and ectopically expressed in *Arabidopsis*; this residue mediates ethylene binding to ethylene receptors, is a candidate ligand for the Cu(I) cofactor, causes the loss of ethylene binding and suppresses the sequential ethylene response (Schaller et al., 1995; Wilkinson et al., 1997; Rodriguez et al., 1999). Eighty independent 35S::*OnETR1-C65Y* transgenic *Arabidopsis* plants were obtained. Sixty-four *OnETR1-C65Y* plants were phenotypically indistinguishable from wild-type plants, whereas the other 16 plants showed a delayed flower senescence phenotype (Figures 4A,B). Flower organs were senescent and abscised at positions 3–4 in wild-type plants (Figures 4E,F) and in 35S::*OnETR1 Arabidopsis* (Figures 4D,F). In *etr1*-*1* mutant plants, senescence was delayed until position 12 (Figures 4A,C,F). In 35S::*OnETR1*-*C65Y* transgenic *Arabidopsis*, senescence was delayed until position 8 (Figures 4A,B,F). To examine whether the ethylene response was affected by 35S::*OnETR1*-*C65Y*, the expression of downstream genes in the ethylene response, such as ethylene response DNA-binding factor *1-4* (*EDF1*-*4*) and ethylene response factor 1 (*ERF1*; Stepanova and Alonso, 2005; Chen et al., 2021), was analyzed in 35S::*OnETR1*-*C65Y Arabidopsis* that showed high expression of *OnETR1*-*C65Y* (Figure 4G). The results indicated that the expression of *EDF1-4* and *ERF1* was significantly downregulated in 35S::*OnETR1*-*C65Y Arabidopsis* (Figure 4H). In the *etr1*-*1* mutant, only *EDF1*, *EDF4*, and *ERF1* were downregulated (Figure 4H). These results indicated that the delayed flower senescence in 35S::*OnETR1*-*C65Y Arabidopsis* was mainly due to the inhibition of the ethylene signaling pathway.

### 35S::*OnETR1*-*C65Y Arabidopsis* Is Insensitive to Ethylene Treatment

**Figure 5:**
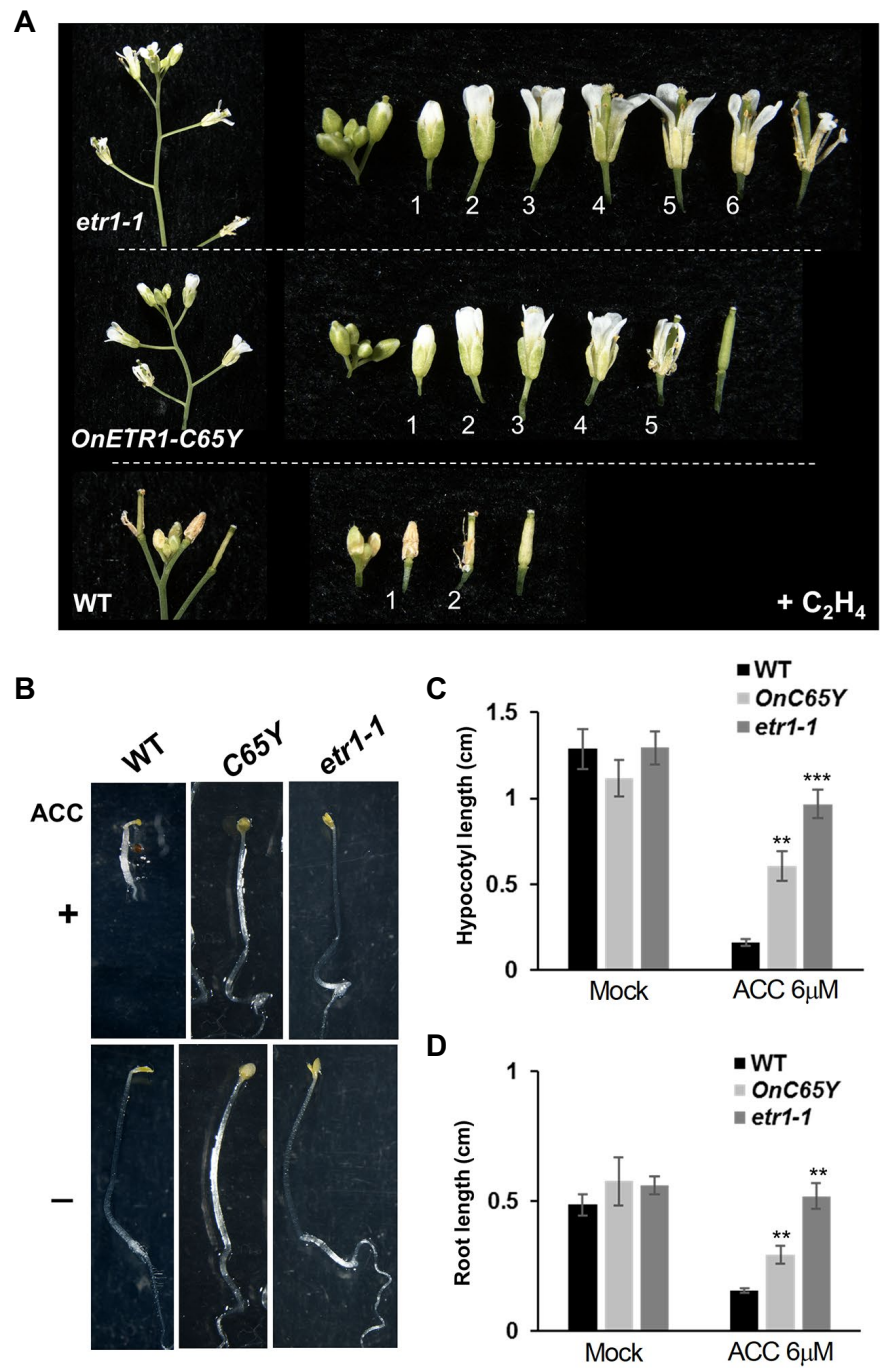
Ethylene treatment and triple response assay in 35S::*OnETR1*-*C65Y* and *etr1-1 Arabidopsis*. **(A)** A comparison of *etr1-1* (top), 35S::*OnETR1*-*C65Y* (middle), and wild-type (bottom) flowers exposed to ethylene (+C_2_H_4_). In the wild type, the flowers were senescent at position 1, whereas *etr1-1* and 35S::*OnETR1*-*C65Y* flowers were not senescent at positions 5–6 after ethylene treatment. **(B)** WT, 35S::*OnETR1-C65Y* (*C65Y*), and *etr1-1 Arabidopsis* seedlings were grown on MS plates in the presence (+, top row) or absence (−, bottom row) of 6 μM 1-aminocyclopropane-1 carboxylic acid (ACC), the ethylene precursor, for 7 days in the dark. Wild-type seedlings showed apical hooks and short hypocotyls and roots after ACC treatment. Triple response phenotypes were not observed in the *etr1-1* or 35S::*OnETR1-C65Y* seedlings after ACC treatment. **(C,D)** Measurement of hypocotyl **(C)** and root **(D)** length in wild-type, 35S::*OnETR1-C65Y* (*C65Y*) and *etr1-1 Arabidopsis* seedlings in the presence (ACC 6 μM) or absence (Mock) of ACC. Values are the average lengths (mean ± SD) of >15 hypocotyls or roots. The error bars represent SD. The asterisks indicate a significant difference from the WT value (^**^p < 0.01 and ^***^p < 0.001). Statistical analyses were conducted using two-tailed Student’s *t*-tests.

To further examine the ethylene response in *OnETR1*-*C65Y Arabidopsis*, 35S::*OnETR1*-*C65Y*, *etr1-1*, and wild-type *Arabidopsis* were exposed to air containing 6 ppm ethylene for 3 days. In the presence of ethylene, the perianth organs senesced and abscised early (at position 1) in wild-type *Arabidopsis* (Figure 5A, bottom). In contrast, in the presence of ethylene, the perianth organs of the 35S::*OnETR1*-*C65Y* and *etr1-1* flowers were turgid and not senescent (Figure 5A, middle and top). These results indicated that the delayed senescence/abscission of the flower organs in 35S::*OnETR1*-*C65Y Arabidopsis* was due to ethylene insensitivity, similar to that in *etr1-1* mutants.

### 35S::*OnETR1-C65Y Arabidopsis* Lacks the Triple Response Phenotype

The application of ethylene to etiolated seedlings results in the triple response, including a curvature of the apical hook and a shortening and thickening of the hypocotyls and roots (Guzman and Ecker, 1990; Bakshi et al., 2015). To confirm the ethylene insensitivity of 35S::*OnETR1-C65Y* plants, the triple response was analyzed in 35S::*OnETR1-C65Y*, *etr1-1*, and wild-type seedlings by growing seedlings in the presence or absence of 6 M ACC, the ethylene precursor. Wild-type seedlings showed apical hooks and extremely short hypocotyls and roots upon ACC treatment (Figures 5B–D). In contrast, 35S::*OnETR1-C65Y* and *etr1-1* seedlings showed no obvious apical hooks and produced longer hypocotyls and roots than did wild-type seedlings after ACC treatment (Figures 5B–D). The lack of a triple response in 35S::*OnETR1-C65Y* seedlings, similar to that in *etr1-1* seedlings, further confirmed that *OnETR1-C65Y* could cause ethylene insensitivity and thus repress ethylene signal transduction in *Arabidopsis*.

### Delayed Leaf Senescence in 35S::*OnETR1-C65Y Arabidopsis* Was Insensitive to ACC and JA Treatment

**Figure 6:**
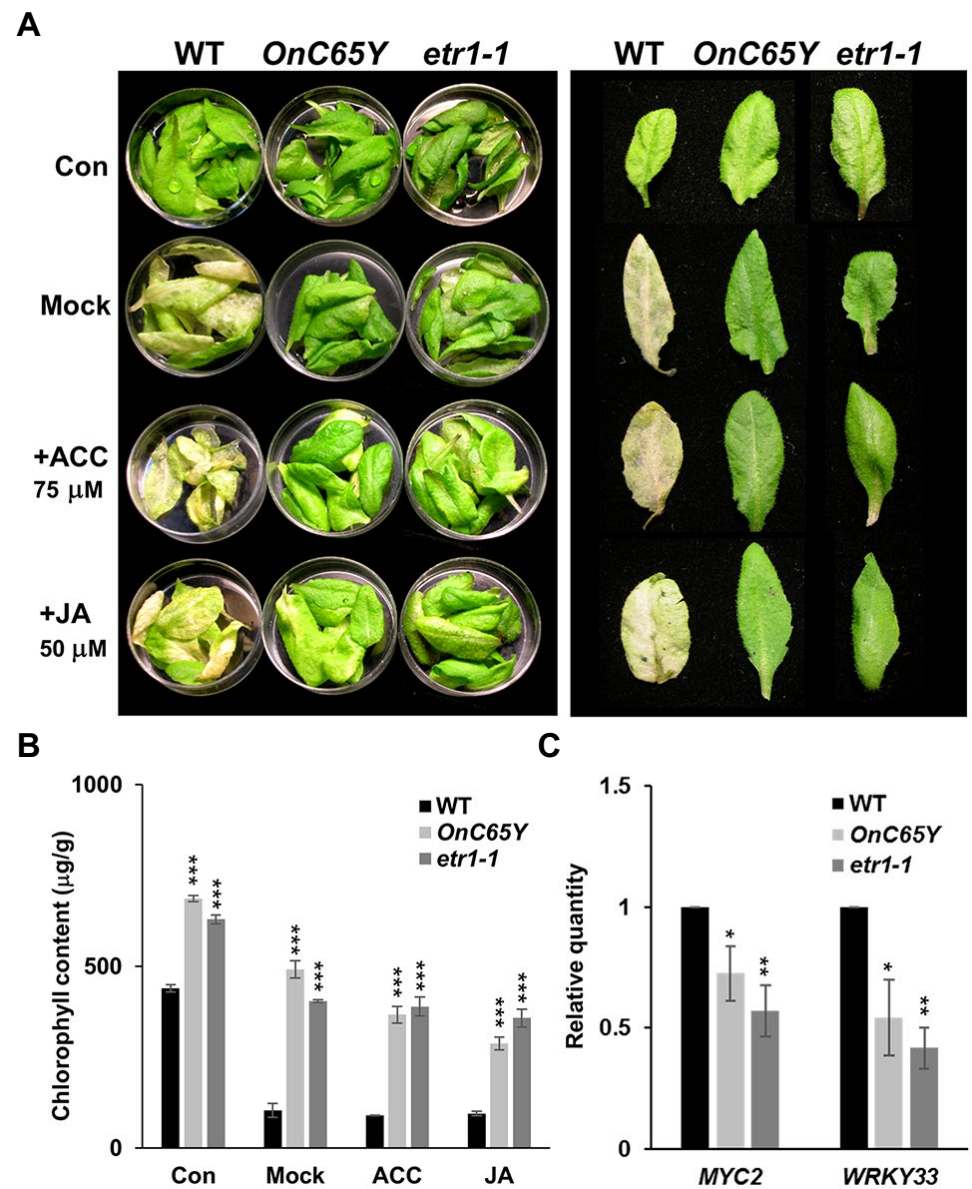
Assay of the effect ACC and jasmonic acid (JA) treatments on detached leaf senescence in 35S::*OnETR1-C65Y* and *etr1-1 Arabidopsis*. **(A)** Phenotypes of detached leaves from WT, 35S::*OnETR1*-*C65Y* (*OnC65Y*) and *etr1*-*1 Arabidopsis* treated with water (Mock), ACC or JA in the dark for 4 days. The control (con) did not receive a dark or ACC/JA treatment. The left panel shows the detached leaves in Petri dishes, whereas the right panel shows a single detached leaf. Detached wild-type leaves were clearly senescent, whereas detached 35S::*OnETR1*-*C65Y* and *etr1*-*1* leaves were still green and showed no sign of senescence after ACC or JA treatment. **(B)** Measurement of the chlorophyll contents of the detached leaves from **(A)**. The error bars represent SD. The asterisks indicate a significant difference from the WT value (^***^p < 0.001). Statistical analyses were conducted using two-tailed Student’s *t*-tests. **(C)** Detections of *MYC2* and *WRKY33* gene expression *via* real-time quantitative PCR. Total RNA isolated from 35S::*OnETR1-C65Y*, *etr1-1* and wild-type *Arabidopsis* was used as a template to detect the expression of *MYC2* and *WRKY33*. The transcript levels of these genes were determined using two to three replicates and were normalized to *UBQ10*. The expression of each gene in the transgenic plants is given relative to that of the wild-type plant, which was set at 1. The error bars represent SDs. The asterisks indicate a significant difference from the WT value (^*^p < 0.05 and ^**^p < 0.01). Statistical analysis was conducting using two-tailed Student’s *t*-tests.

Similar to ethylene, JA has also been demonstrated to promote senescence in *Arabidopsis* (He et al., 2002). To further analyze the effect of ethylene and JA on leaf senescence in 35S::*OnETR1*-*C65Y* plants, detached leaves from 35S::*OnETR1*-*C65Y*, *etr1*-*1* and wild-type plants were treated with ACC and JA in the dark, and the phenotypes were analyzed. The results indicated that the detached leaves of wild-type *Arabidopsis* were yellow and withered in the dark after 4 days (Figure 6A, second row, left) compared to the control (Figure 6A, first row, left). In addition, the wild-type leaves showed ACC- and JA-induced senescence after treatment with ACC or JA in the dark for 4 days (Figure 6A, third and fourth rows, left). In contrast, the detached leaves from 35S::*OnETR1*-*C65Y* and *etr1-1* stayed green without signs of senescence upon treatment with ACC or JA in the dark for 4 days (Figure 6A, second to fourth rows, middle and right). Furthermore, chlorophyll remained significantly higher in the detached leaves from 35S::*OnETR1-C65Y* and *etr1-1* plants than in wild-type plants after dark, ACC or JA treatments (Figure 6B). These results indicated that 35S::*OnETR1-C65Y* could inhibit leaf senescence by repressing the signal transduction of not only ethylene but also JA.

### 35S::*OnETR1-C65Y* Inhibited Senescence by Suppressing *MYC2*/*WRKY33* in JA Signaling

*MYC2* and *WRKY33* act as downstream genes in JA signaling and activate JA responses (Gfeller et al., 2010). Since 35S::*OnETR1*-*C65Y* and *etr1-1* displayed inhibited senescence and JA insensitivity, an analysis of the expression of *MYC2* and *WRKY33* in 35S::*OnETR1*-*C65Y* and *etr1-1* plants was performed. The results clearly indicated that *MYC2* and *WRKY33* expression was significantly downregulated in 35S::*OnETR1*-*C65Y* and *etr1-1* plants (Figure 6C). This result supported the idea that delayed leaf and flower senescence by 35S::*OnETR1*-*C65Y* was also due to the suppression of JA signaling through the downregulation of *MYC2* and *WRKY33* expression.

### 35S::*OnETR1-C65Y* Caused Male Sterility Due to the Production of Indehiscent Anthers

**Figure 7:**
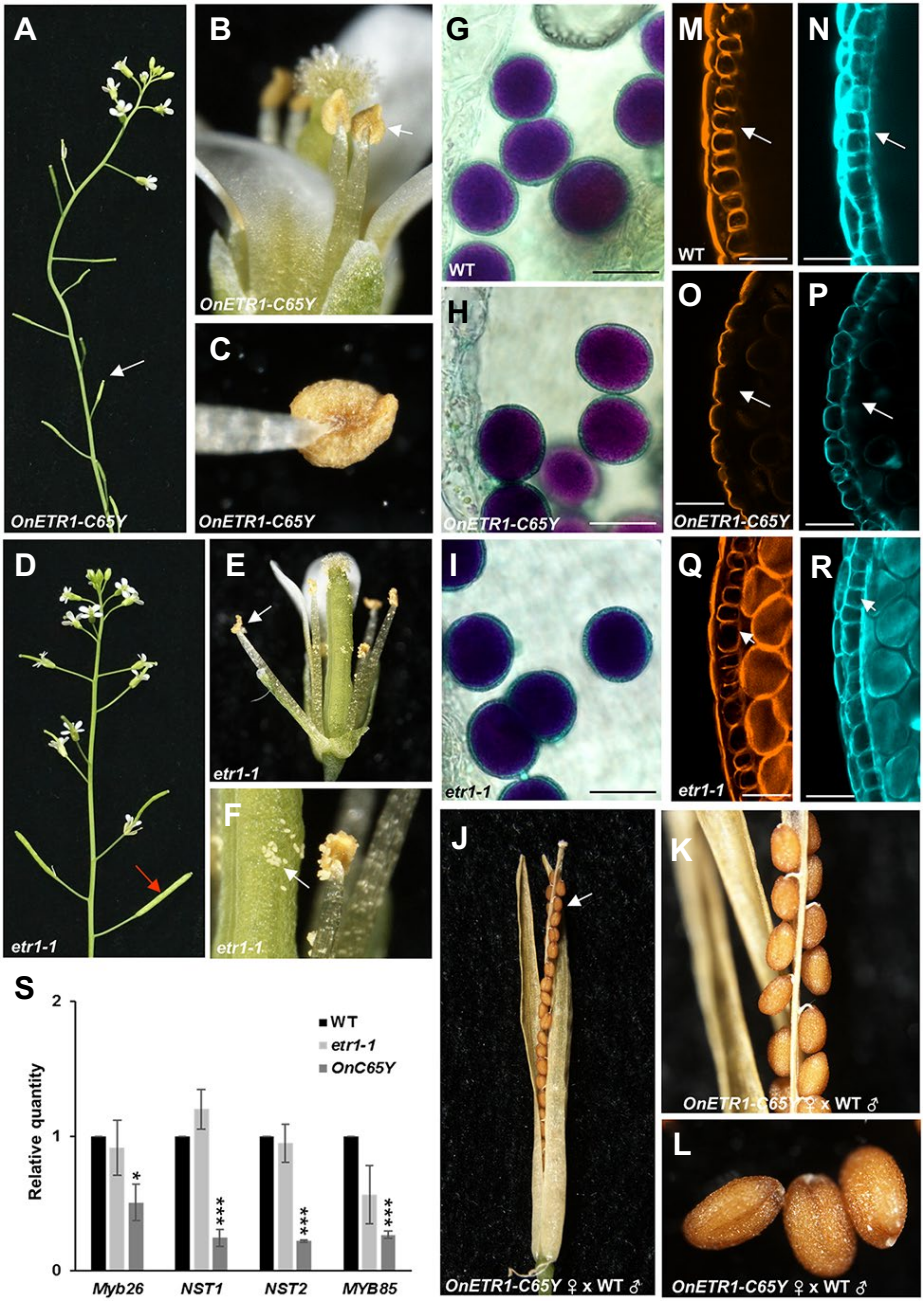
Analysis of the indehiscent anther phenotype and the detection of gene expression in 35S::*OnETR1*-*C65Y Arabidopsis*. **(A)** Inflorescence of a 35S::*OnETR1*-*C65Y* plant showing short siliques (arrow). **(B)** A 35S::*OnETR1*-*C65Y* flower with an indehiscent anther (arrow). **(C)** Close-up view of an indehiscent anther from **(B)**. **(D)** Inflorescence of an *etr1-1* mutant with elongated siliques (arrow). **(E)** An *etr1-1* flower with a dehiscent anther (arrow). **(F)** Close-up view of a dehiscent anther and released pollen (arrow) from **(E)**. **(G–I)** Pollen grain viability analysis of 35S::*OnETR1*-*C65Y* male-sterile anthers *via* Alexander staining. Wild-type **(G)**, 35S::*OnETR1*-*C65Y* **(H)**, and *etr1-1* **(I)** *Arabidopsis* had similar viable pollen grains with similar shapes. Bar = 20 μm. **(J)** The normal elongated silique and normal seeds (arrow) of a 35S::*OnETR1*-*C65Y* plant after pollination with wild-type pollen. **(K,L)** Close up of the normal seeds in the silique from **(J)**. **(M–R)** CLSM of anther cell walls in wild-type **(M,N)**, 35S::*OnETR1*-*C65Y* **(O,P)**, and *etr1-1* **(Q,R)** *Arabidopsis*. The anthers were double stained with auramine O for lignin, indicated in orange **(M,O,Q)**, and with calcofluor white for cellulose, indicated cyan **(N,P,R)**. Secondary thickening is visible in the endothecium (arrowed) of the wild-type **(M,N)** and *etr1-1* **(Q,R)** anthers but is absent in 35S::*OnETR1*-*C65Y* **(O,P)** anthers (arrowed). Bar = 25 μm. **(S)** mRNA levels as determined *via* real-time quantitative PCR. Total RNA isolated from wild-type, *etr1-1* and 35S::*OnETR1*-*C65Y* plants was used as a template to detect the expression of *MYB26*, *NST1/2*, and *MYB85*. The transcript levels of these genes were determined using two to three replicates and were normalized to *UBQ10*. The expression of each gene in the transgenic plants is given relative to that of the wild-type plant, which was set at 1. The error bars represent SDs. The asterisks indicate a significant difference from the WT value (^*^p < 0.05 and ^***^p < 0.001). Statistical analysis was conducting using two-tailed Student’s *t*-tests.

In addition to the delay in flower senescence, 35S::*OnETR1-C65Y* also caused male sterility, with the production of indehiscent anthers and unelongated siliques (Figures 7A–C). Interestingly, this indehiscent anther phenotype was not observed in *etr1-1* mutants, which produced dehiscent anthers and elongated siliques (Figures 7D–F). When Alexander’s staining was used, the anthers of 35S::*OnETR1-C65Y Arabidopsis* contained viable pollen grains (Figure 7H) that closely resembled those in wild-type (Figure 7G) and *etr1-1* (Figure 7I) plants. To further examine pistil activity, wild-type pollen was crossed with stigmas of sterile 35S::*OnETR1-C65Y Arabidopsis*. The siliques exhibited normal elongation and produced normal seeds after cross-pollination (Figures 7J–L). The results indicated that the sterility observed in 35S::*OnETR1-C65Y* plants was associated with the production of indehiscent anthers.

### 35S::*OnETR1-C65Y* Caused a Failure in Secondary Cell Wall Thickening and the Downregulation of *MYB26*, *NST1*, *NST2*, and *MYB85*

To analyze the anther indehiscence phenotype in 35S::*OnETR1-C65Y* plants, the secondary cell wall thickness of the endothecium was further examined by double staining with auramine O for lignin, which is indicated in orange (Figures 7M,O,Q), and with calcofluor white for cellulose, which is indicated in cyan (Figures 7N,P,R). The results indicated that the secondary cell wall thickened normally in wild-type anthers, with striated lignin formation in the endothecium (Figures 7M,N). In contrast, 35S::*OnETR1*-*C65Y* plants could not develop secondary cell wall thickening, and lignin formation was absent in the endothecium (Figures 7O,P). In contrast to 35S::*OnETR1*-*C65Y* plants, in *etr1-1* anthers, normal secondary cell wall thickening was also observed, with striated lignin formation in the endothecium (Figures 7Q,R). When the expression of *MYB26*, *MYB85*, and *NST1*/2, which have been thought to regulate secondary thickening in anther endothecium (Mitsuda et al., 2005; Zhong et al., 2008), was analyzed, all four genes were clearly downregulated in 35S::*OnETR1*-*C65Y* plants and were not affected in *etr1-1* mutants (Figure 7S). These results indicated that 35S::*OnETR1*-*C65Y* caused a failure in secondary cell wall thickening due to the downregulation of *MYB26/MYB85*/*NST1*/2, which led to the production of indehiscent anthers.

## Discussion

The regulatory role of ethylene in plant senescence has been well characterized, and ethylene-induced senescence is a considerable physiological problem in cut *Oncidium* flowers. Removing the pollinia caps of *Oncidium* increases ethylene production and leads to flower senescence during harvest and transport (Huang et al., 2007). Thus, the control of ethylene synthesis and signal transduction genes in *Oncidium* is important. In this study, we identified *OnACS12*, an ethylene synthesis gene, and *OnETR1*, an ethylene signal transduction gene, from *Oncidium* and functionally characterized them in transgenic *Arabidopsis*.

**Figure 8:**
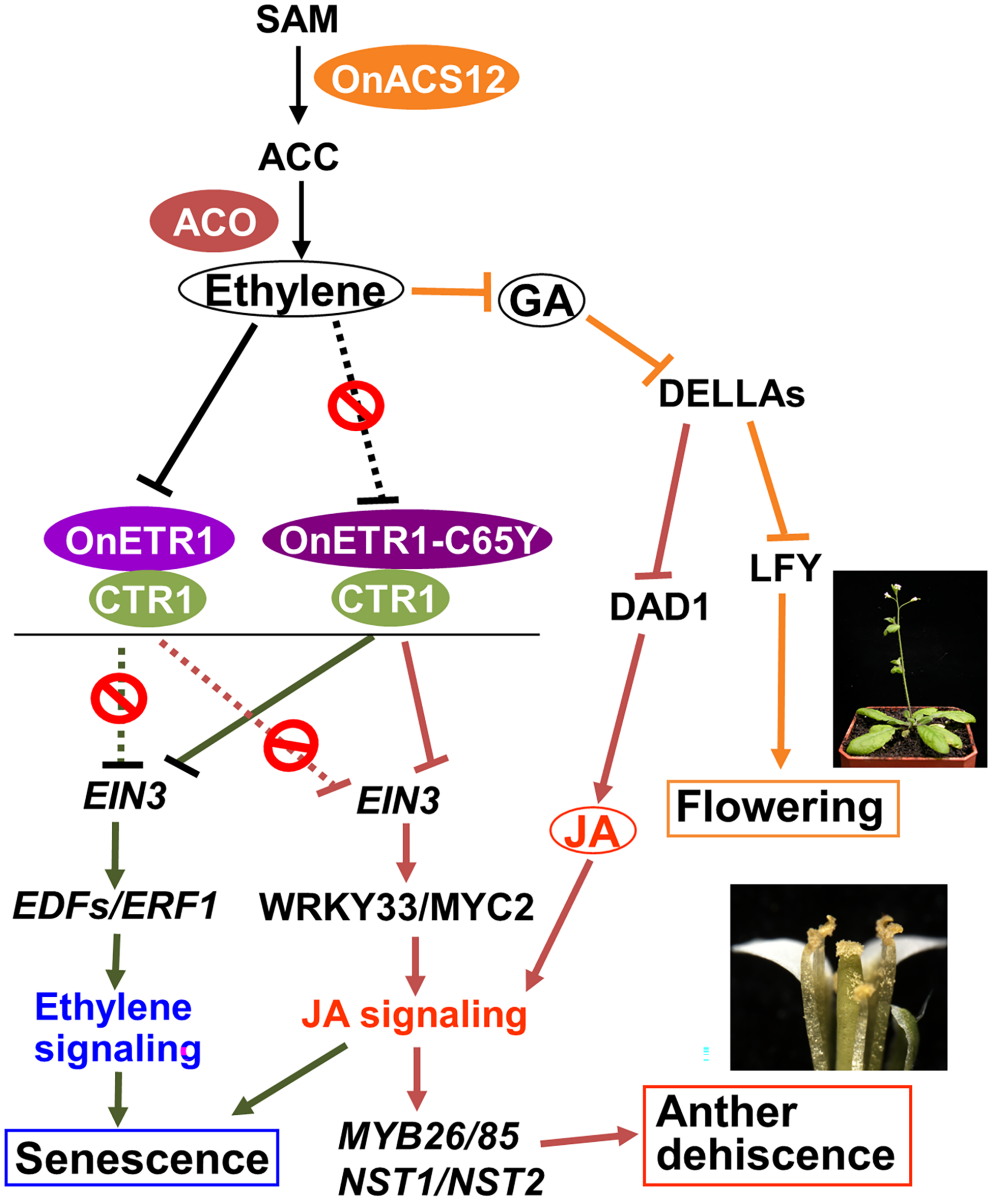
Model for the function of *OnACS12* and *OnETR1*-*C65Y* in regulating ethylene, GA-DELLA and JA signaling in *Arabidopsis*. The ectopic expression of *Oncidium OnACS12* delayed flowering and produced indehiscent anthers in *Arabidopsis* through an increase (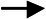) in *ACC* and ethylene production, which influenced GA–DELLA signaling by suppressing (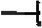) GA production and activating DELLA activity. The increased DELLA activity sequentially suppressed (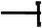) the expression of the flowering time gene *LFY* and caused late flowering phenotypes. The increased DELLA activity also suppressed (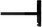) the expression of the JA biosynthesis gene *DAD1*, which caused a decrease in JA activity, the suppression of *MYB26*, *NST1/2*, and *MYB85* expression and the failure of secondary thickening in the endothecium, resulting in the production of indehiscent anthers. In wild-type plants, *Oncidium OnETR1* can bind to and be suppressed (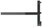) by ethylene, resulting in the inability (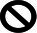) to activate CTR1 to (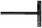) suppress positive regulators (i.e., *EIN3*) and downstream genes (*EDFs* and *ERF1*) of the ethylene response, as well as the downstream JA signaling genes *MYC2* and *WRKY33*. This caused a normal ethylene and JA response and led to leaf/flower senescence and anther dehiscence, respectively. The ectopic expression of *OnETR1-C65Y* blocked (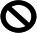) normal ethylene binding and sequentially activated CTR1 to suppress (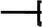) the positive regulators (i.e., *EIN3*) and downstream genes (*EDFs* and *ERF1*) of the ethylene response, as well as the downstream JA signaling genes *MYC2* and *WRKY33*, resulting in delayed leaf/flower senescence and indehiscent anthers in *Arabidopsis*.

The ectopic expression of *OnACS12* in *Arabidopsis* can cause increased ethylene production and a late flowering phenotype. Ethylene regulates flowering time *via* GA–DELLA signaling (Achard et al., 2007). GA is perceived by GA INSENSITIVE DWARF (GID) 1 and 2, which are soluble nuclear receptors. GIDs can bind to all bioactive gibberellins and interact with DELLAs to enhance the binding of the ubiquitin E3 ligase complex SCF to DELLAs, thereby promoting DELLA degradation *via* the 26S proteasome pathway (Sasaki et al., 2003; Griffiths et al., 2006). The DELLA proteins include five members, *GAI*, *RGA*, and *RGA-LIKE* (*RGL1*, *RGL2*, and *RGL3*); they act as repressors of GA signaling and delay the timing of the floral transition, possibly through the downregulation of *LFY* (Wen and Chang, 2002; Harberd, 2003; Achard et al., 2006; Figure 8). We verified that the late flowering phenotype was correlated with the alteration of GA–DELLA signaling pathways in 35S::*OnACS12 Arabidopsis*. Three GA biosynthetic genes, *CPS*, *KS*, and *GA3ox1*, were downregulated; three DELLA genes, *GAI*, *RGL1*, and *RGL2*, were upregulated; and *LFY* expression was suppressed in 35S::*OnACS12* plants. These results implied that bioactive GA levels were downregulated by decreasing gene transcripts encoding GA biosynthetic enzymes, which caused the accumulation of DELLAs, thus enhancing DELLA activity to suppress the expression of *LFY* and cause a delay in flowering. Thus, the ectopic expression of orchid *OnACS12* extended the vegetative phase of *Arabidopsis* by increasing ethylene production, which sequentially influenced GA–DELLA signaling and the reduction of *LFY* expression.

In addition to delaying flowering, 35S::*OnACS12* also caused male sterility due to anther indehiscence in transgenic *Arabidopsis*. It has been reported that GA can regulate anther dehiscence by repressing DELLA proteins and sequentially activating JA biosynthetic genes such as *DAD1* (Cheng et al., 2009; Peng, 2009; Marciniak and Przedniczek, 2019). JA is thought to play an important role in regulating anther dehiscence (Sanders et al., 2000; Zhao and Ma, 2000; Ishiguro et al., 2001; Scott et al., 2004), and mutations in genes that participate in JA biosynthesis, such as *DAD1* and *OPR3*, cause a similar delay in anther dehiscence (Sanders et al., 1999, 2000; Stintzi and Browse, 2000; Scott et al., 2004). GA also regulates anther dehiscence in yellow lupine (*Lupinus luteus* L.) by regulating genes involved in controlling anther structure such as secondary thickening in the endothecium (Marciniak and Przedniczek, 2021). Since we have shown that 35S::*OnACS12* can suppress GA levels and upregulate the accumulation of DELLA proteins, the exploration of the relationship between 35S::*OnACS12* and JA biosynthesis was necessary. Unsurprisingly, the expression of the key JA biosynthetic gene *DAD1* was downregulated in 35S::*OnACS12 Arabidopsis*. Thus, the enhancement of DELLA activity through the suppression of GA activity not only caused late flowering by suppressing *LFY* expression but also caused anther indehiscence by suppressing *DAD1* expression and JA activity in 35S::*OnACS12 Arabidopsis* (Figure 8). In addition to the downregulation of the JA biosynthetic gene *DAD1*, the expression of *MYB26* and *NST1*/*NST2*, which are thought to regulate secondary thickening in the anther endothecium (Mitsuda et al., 2005; Zhong et al., 2008), was clearly downregulated in 35S::*OnACS12 Arabidopsis*. The *Arabidopsis myb26* mutant exhibits anther indehiscence, and secondary thickening does not occur in the endothecium (Yang et al., 2007). *NST1* and *NST2*, which are thought to act downstream of *MYB26*, are associated with secondary cell wall thickening and endothecium lignification. Thus, once ectopically expressed in *Arabidopsis*, orchid *OnACS12* can increase ethylene production, which affects GA–DELLA signaling and lowers JA activity, resulting in the failure of secondary thickening in the anther endothecium and the production of an anther indehiscence phenotype.

In this study of the *Oncidium OnETR1* gene, the dominant-negative mutation *OnETR1-C65Y*, which could potentially prevent ethylene binding and block ethylene signaling (Wilkinson et al., 1997; Rodriguez et al., 1999), was ectopically expressed in *Arabidopsis*. Unsurprisingly, a significant delay in flower senescence was observed in 35S::*OnETR1-C65Y Arabidopsis*, which was similar to the phenotype observed in the *etr1-1* mutant and in 35S::*AtETR1-C65Y* transgenic tomato, petunia (Wilkinson et al., 1997) and tobacco (Yang et al., 2008). Similar to the *etr1-1* mutant, 35S::*OnETR1-C65Y* flowers also showed insensitivity to ethylene treatment, and seedlings lacked the triple response phenotype after exposure to an external supply of ACC. In addition, detached leaves from 35S::*OnETR1-C65Y* and *etr1-1* did not show senescence after ACC treatment. Thus, our results demonstrated that orchid *OnETR1* has the same ability as *Arabidopsis AtETR1* to act as an ethylene receptor in transgenic *Arabidopsis*. The ectopic expression of *OnETR1-C65Y* in *Arabidopsis* altered ethylene binding and suppressed ethylene signaling, which downregulated the expression of the ethylene signal transduction genes *EDFs* and *ERF1* (Stepanova and Alonso, 2005; Chen et al., 2021) and resulted in an ethylene insensitive dominant-negative mutant phenotype, such as a delay in flower/leaf senescence (Figure 8).

One interesting result is that detached leaves from 35S::*OnETR1-C65Y* and the *etr1-1* mutant also showed JA insensitivity since the detached leaves did not senesce after JA treatment. JA plays a role in leaf senescence (Ueda and Kato, 1980), and EIN3 in ethylene signaling is required for JA-induced leaf senescence (Li et al., 2013). Thus, 35S::*OnETR1-C65Y* should also be able to suppress JA signaling due to the suppression of ethylene signaling and result in ethylene/JA insensitivity. This assumption was further supported by the downregulation of the downstream JA signaling genes *MYC2* and *WRKY33* (Gfeller et al., 2010) in 35S::*OnETR1-C65Y Arabidopsis*. Thus, our results demonstrated that ethylene signaling can regulate some ethylene responses, such as senescence, through the activation of JA signal transduction in plants (Figure 8). This finding could be used to explain the additional indehiscent anther phenotype observed in transgenic 35S::*OnETR1-C65Y Arabidopsis*. As described above, JA can regulate anther dehiscence (Sanders et al., 2000; Zhao and Ma, 2000; Ishiguro et al., 2001; Scott et al., 2004) since mutations in genes that participate not only in JA biosynthesis (Sanders et al., 1999, 2000; Stintzi and Browse, 2000; Scott et al., 2004) but also in JA signaling, such as *coronatine insensitive 1* (*coi1*), cause similar anther indehiscence phenotypes (Feys et al., 1994; Xie et al., 1998). It is thus reasonable to propose that 35S::*OnETR1-C65Y* suppressed ethylene signaling, which downregulated the JA signaling genes *MYC2* and *WRKY33* and resulted in the suppression of *MYB26/MYB85/NST1/2* expression and the anther indehiscence phenotype, as seen in our results (Figure 8). In contrast, in wild-type plants, the normal binding of ethylene to AtETR1/OnETR1 activated ethylene signaling, which activated the expression of the JA signal transduction genes *MYC2*/*WRKY33* and *MYB26/MYB85/NST1/2* and resulted in normal anther dehiscence. This result further confirmed the crosstalk between ethylene and JA in regulating both flower/leaf senescence and anther dehiscence in plants (Figure 8).

Notably, the anther indehiscence phenotype was not seen in *etr1-1* mutants. Despite a similar delayed flower senescence phenotype as that of 35S::*OnETR1-C65Y* plants, *etr1-1* mutants exhibited normal anther dehiscence and pollen release. This phenotype was confirmed based on the normal expression of *MYB26/MYB85/NST1/2* in *etr1-1* mutant flowers. One possible explanation is that *ETR1* does not normally function or has a smaller role among ethylene receptors during anther development. In *etr1-1* mutants, other ethylene receptors (ERS1, ETR2, ERS2, and EIN4) may still interact with ethylene to sequentially activate ethylene signaling and responses, resulting in the activation of JA signaling and normal anther dehiscence. In 35S::*OnETR1-C65Y* plants, the ectopic expression of *OnETR1-C65Y* during anther development generated dominant-negative mutations for all ethylene receptors, which blocked the binding of ethylene and caused the sequential suppression of ethylene signaling/response and JA signaling, resulting in the production of indehiscent anthers.

In summary, the *Oncidium* ethylene synthesis gene *OnACS12* and the ethylene signal transduction gene *OnETR1* were functionally characterized in transgenic *Arabidopsis*. We found that *OnACS12* and *OnETR1* could perform their functions in the ethylene response by interacting with the plant hormones GA and JA. The ectopic expression of *OnACS12* could delay flowering *via* the regulation of GA–DELLA signaling pathways and *LFY* expression. *OnETR1-C65Y* could regulate flower/leaf senescence through the suppression of ethylene and JA signaling. Most interestingly, both *OnACS12* and *OnETR1-C65Y* could regulate anther dehiscence by downregulating the JA biosynthetic gene *DAD1* or by suppressing JA signaling, respectively. The characterization of the *OnACS12* and *OnETR1* genes in this study not only provides useful data for understanding the functions of *ACS* and *ETR1* orthologs in regulating ethylene/GA/JA responses during various plant developmental processes but also provides a feasible future strategy to control flower senescence in *Oncidium* orchids by introducing a dominant-negative *OnETR1-C65Y* mutation or to generate male sterility for valuable crops through the ectopic expression of either *OnACS12* or *OnETR1-C65Y*.

## Data Availability Statement

The original contributions presented in the study are included in the article/**Supplementary Material**, further inquiries can be directed to the corresponding author.

## Author Contributions

C-HY developed the overall strategy, designed experiments, coordinated the project, and prepared and revised the manuscript. T-HH performed gene cloning, transgenic plants, and gene expression experiments. W-HH performed Alexander, Calcofluor White, and Auramine O staining. W-TM performed orchid gene expression analyses. All authors contributed to the article and approved the submitted version.

## Funding

This work was supported by grants to C-HY from the Ministry of Science and Technology, Taiwan, grant number: MOST 103-2313-B-005-001-MY3 and MOST 106-2321-B-005-010. This work was also financially supported (in part) by the Advanced Plant Biotechnology Center from The Featured Areas Research Center Program within the framework of the Higher Education Sprout Project by the Ministry of Education (MOE) in Taiwan.

## Conflict of Interest

The authors declare that the research was conducted in the absence of any commercial or financial relationships that could be construed as a potential conflict of interest.

## Publisher’s Note

All claims expressed in this article are solely those of the authors and do not necessarily represent those of their affiliated organizations, or those of the publisher, the editors and the reviewers. Any product that may be evaluated in this article, or claim that may be made by its manufacturer, is not guaranteed or endorsed by the publisher.

## Supplementary Material

The Supplementary Material for this article can be found online at: https://www.frontiersin.org/articles/10.3389/fpls.2022.785441/full#supplementary-material

## Footnotes

^1^ http://www.bio-rad.com/
