# Supplementary material for: The Oncidium Ethylene Synthesis Gene Oncidium 1-Aminocyclopropane-1 Carboxylic Acid Synthase 12 and Ethylene Receptor Gene Oncidium ETR1 Affect GA–DELLA and Jasmonic Acid Signaling in Regulating Flowering Time, Anther Dehiscence, and Flower Senescence in Arabidopsis
Source: Front Plant Sci. 2022 Apr 1;13:785441. doi: 10.3389/fpls.2022.785441 (PMC9011138; doi:10.3389/fpls.2022.785441)
Supplement: Supplementary file 1 [file Data_Sheet_1.PDF]

Huang_S Fig.1

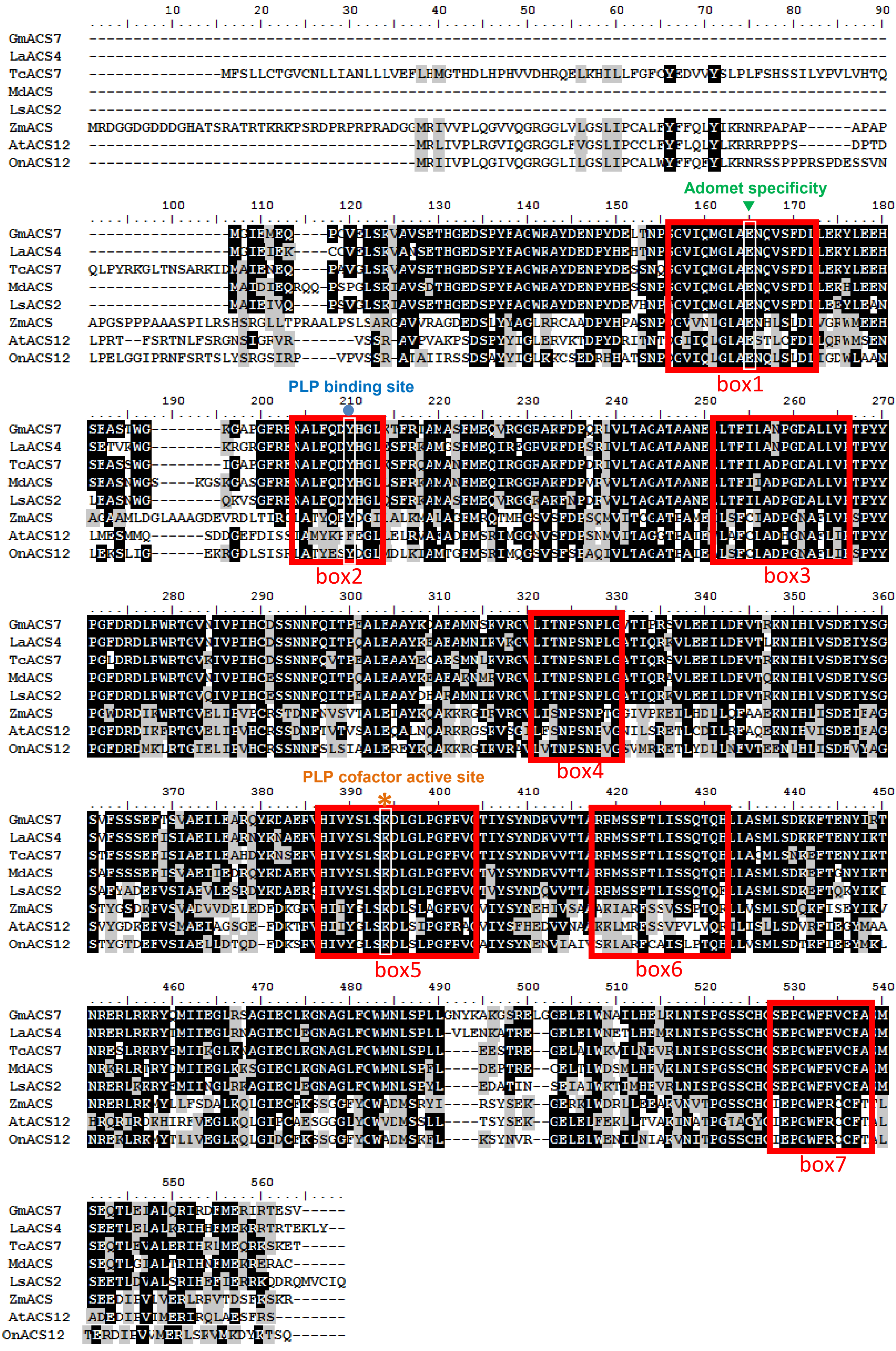
GmACS7
LaACS4
TcACS7
MdACS
LsACS2
ZmACS
AtACS12
OnACS12
Adomet specificity
PLP binding site
PLP cofactor active site
box1
box2
box3
box4
box5
box6
box7

Supplementary Figure 1 Amino acid sequences alignment of OnACS12 and related ACS proteins.

Alignment of the amino acid sequences of OnACS12 and related ACS proteins GmACS7 (*Glycine* max), LaACS4 (*Lupinus* albus), TcACS7 (*Theobroma* cacao), MdACS (*Malus* domestica), LsACS2 (*Lactuca* sativa), ZmACS (*Zea* mays) and AtACS12 (*Arabidopsis thaliana*). The conserved glutamate residue (E) marked with a triangle in box 1 is involved in adomet specificity (Mccarthy et al. 2001). The tyrosine (Y) residue marked with a full circle in box 2 is involved in the PLP-binding site (Capitani et al. 1999). The lysine (K) residue marked with an asterisk in box 5 is involved in the PLP cofactor active site. The seven boxes region (in red) represented high conserved amino acid domain among ACS proteins (Yamagami et al. 2003). The dark highlights indicate identical residues and gray highlights indicate similar residues. Dashes were introduced to improve alignment. The amino acid sequences were aligned by the BioEdit program using ClustalW Multiple Alignment.

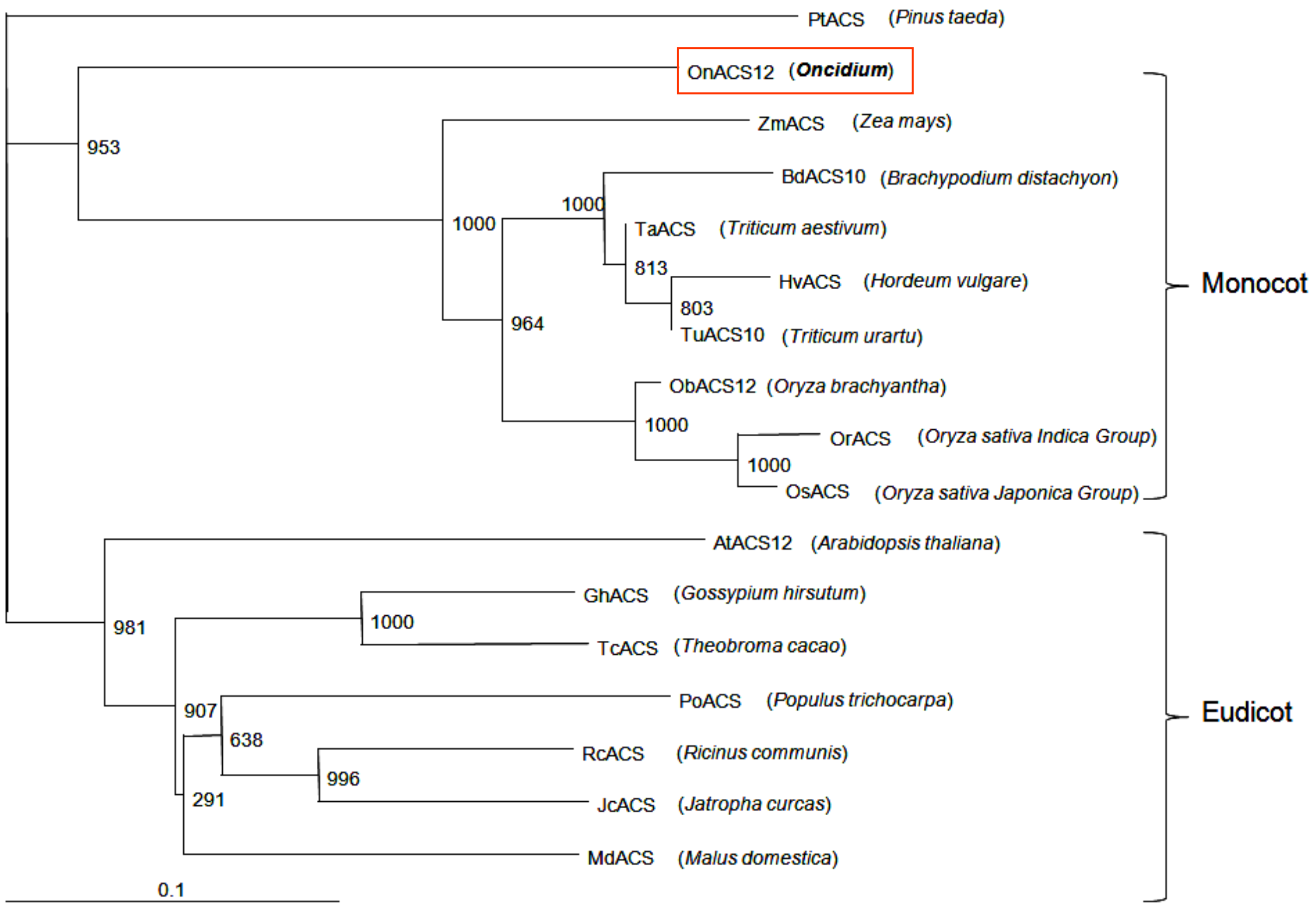

Supplementary Figure 2 Phylogenetic analysis of ACS proteins.
Amino acid sequences of ACS proteins were obtained via the National Center for Biotechnology Information server (http://www.ncbi.nlm.nih.gov/). Names of the plant species of each *ACS* gene are listed behind the protein names. *Oncidium* OnACS12 was red boxed. The multiple sequence alignment was performed by using ClustalW of DNA Data Bank of Japan (http://clustalw.ddbj.nig.ac.jp/top-e.html). The phylogenetic graph was generated by using the TREEVIEW program. Numbers on major branches indicate bootstrap percentages for 1,000 replicate analyses.

Huang_S Fig.3

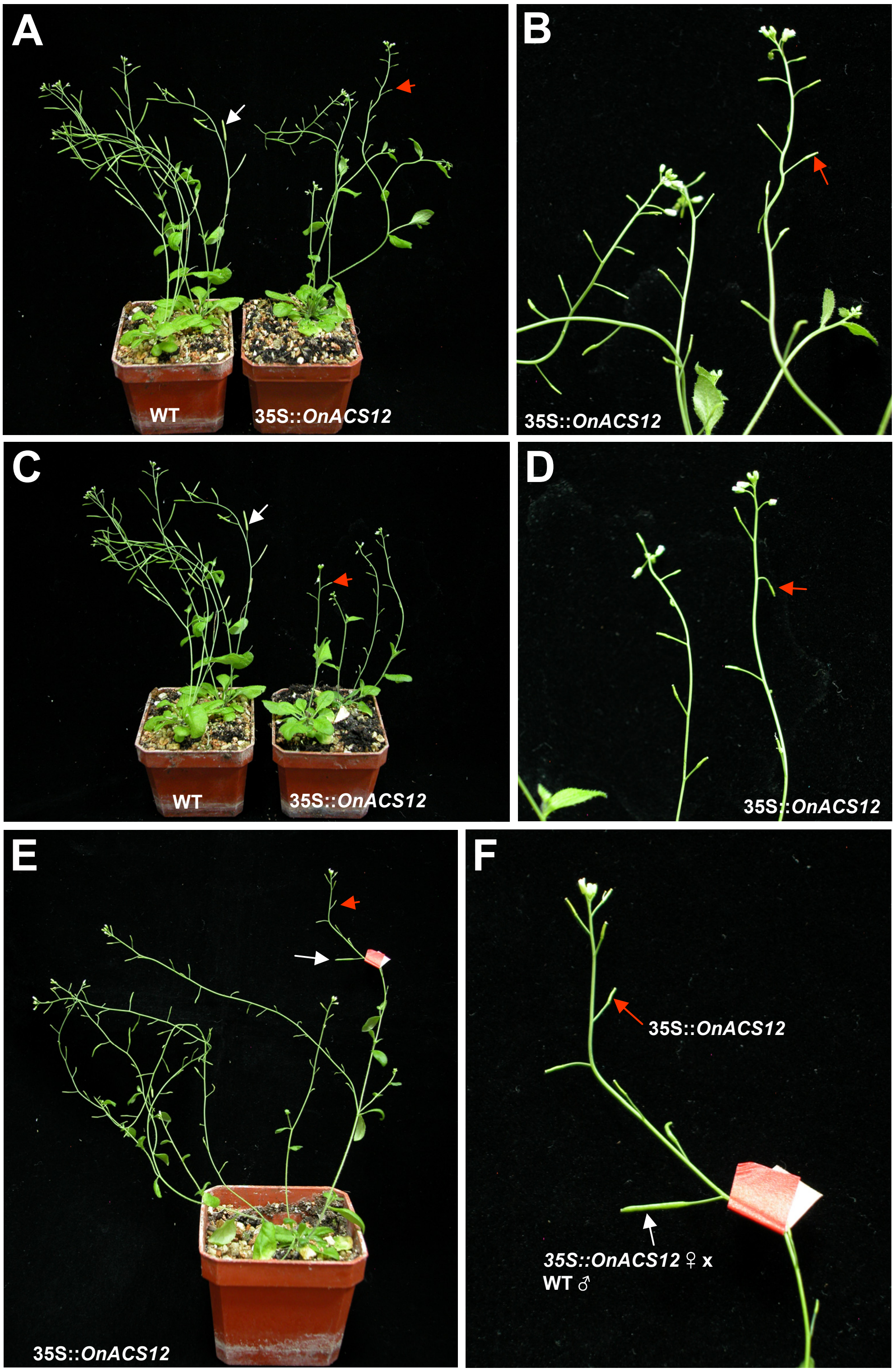
A
WT
35S::*OnACS12*
B
35S::*OnACS12*
C
WT
35S::*OnACS12*
D
35S::*OnACS12*
E
35S::*OnACS12*
F
35S::*OnACS12*
35S::*OnACS12* ♀ x
WT ♂

Supplementary Figure 3 Analysis of the indehiscent anther phenotype in 35S::*OnACS12 Arabidopsis*.

(A) A 52-day-old 35S::*OnACS12* plant produced short siliques (red arrow on the right), whereas the wild-type plant produced normal elongated siliques (white arrow on the left).

(B) Close-up view of the 35S::*OnACS12* inflorescence from **(A)** showing short siliques (red arrow).

(C) Two other 52-day-old 35S::*OnACS12* plants produced short siliques (red arrow on the right), whereas the wild-type plant produced normal elongated siliques (white arrow on the left).

(D) Close-up view of the 35S::*OnACS12* inflorescence from **(C)** showing short siliques (red arrow).

(E) The normal elongated silique of a 35S::*OnACS12* plant after cross-pollination with wild-type pollen (white arrow by the red tape) along with the short 35S::*OnACS12* siliques without pollination (red arrow).

(F) Close-up view of the 35S::*OnACS12* ♀ x WT ♂ elongated silique (white arrow by the red tape) and the short 35S::*OnACS12* siliques (red arrow) from **(E)**.

Huang_S Fig4

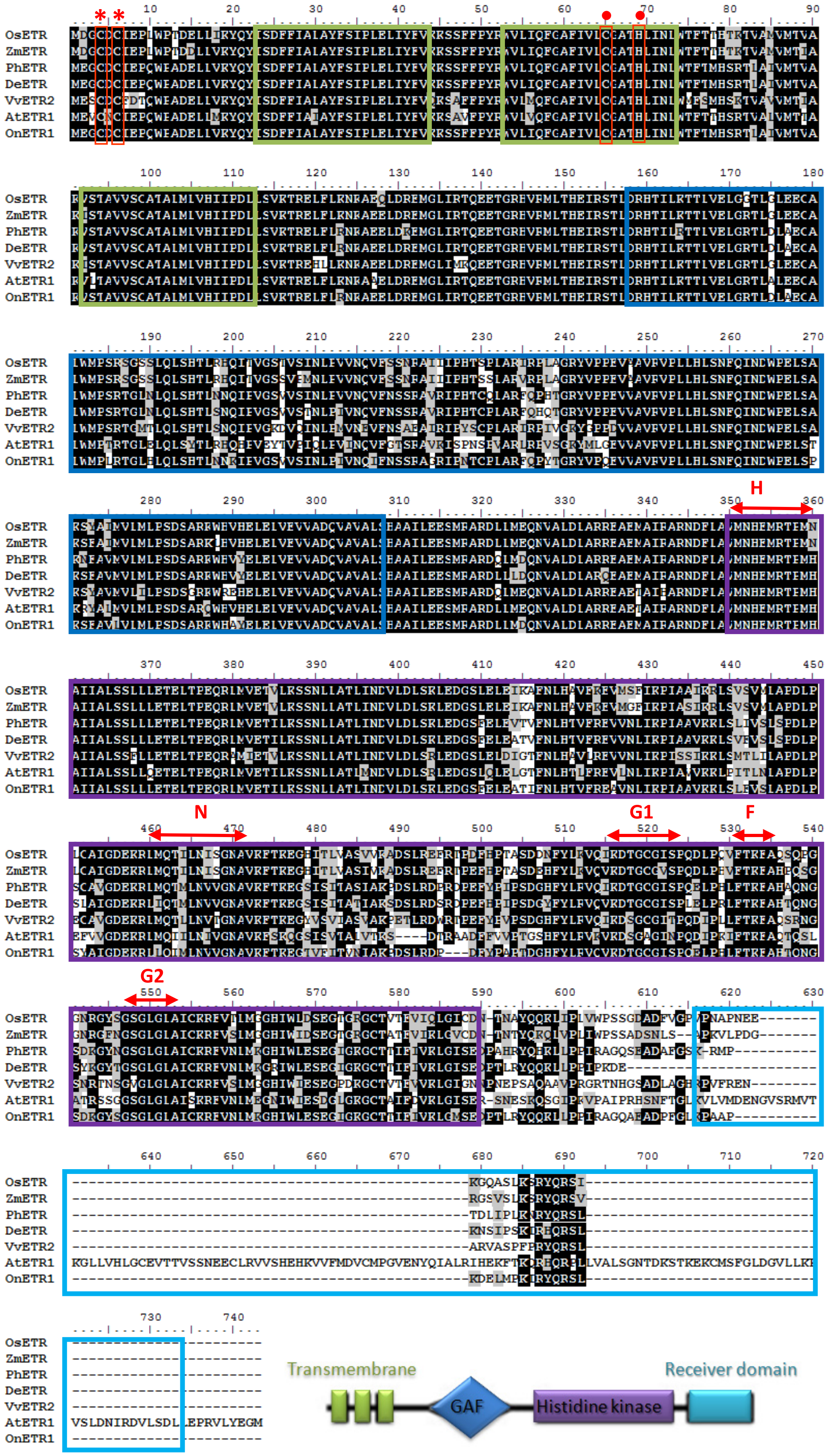
OsETR
ZmETR
PhETR
DeETR
VvETR2
AtETR1
OnETR1
H
N
G1
F
G2
Transmembrane
GAF
Histidine kinase
Receiver domain

Supplementary Figure 4 Amino acid sequences alignment of OnETR1 and related ETR proteins.

Alignment of the deduced amino acid sequences of OnETR1 and related ETR proteins OsETR (*Oryza sativa*), ZmETR (*Zea mays*), PhETR (*Phalaenopsis*), DeETR (*Dendrobium*), VvETR2 (*Vitis vinifera*) and AtETR1 (*Arabidopsis thaliana*). The ethylene binding site composed of three transmembrane domains indicated were in green boxes. The blue box region showed GAF domain. The histidine kinase domain (in the purple box) contained five conserved motifs (under the red arrows) including H, N, G1, F and G2 motifs. The cyan box in C terminal showed putative receiver domain (Parkinson and Kofoid 1992). The two cysteine residues Cys-4 and Cys-6 marked with asterisk are involved in forming disulfide-linked dimer required for receptor function. The two conserved residues Cys-65 and His-69 marked with a full circle in the second transmembrane domain are considered to mediate ethylene binding to ethylene receptor and to be candidate ligands for the Cu(I) cofactor (Schaller and Bleecker 1995). The dark highlights indicate identical residues and gray highlights indicate similar residues. Dashes were introduced to improve alignment. The amino acid sequences were aligned by the BioEdit program using ClustalW Multiple Alignment.

Huang_S Fig.5

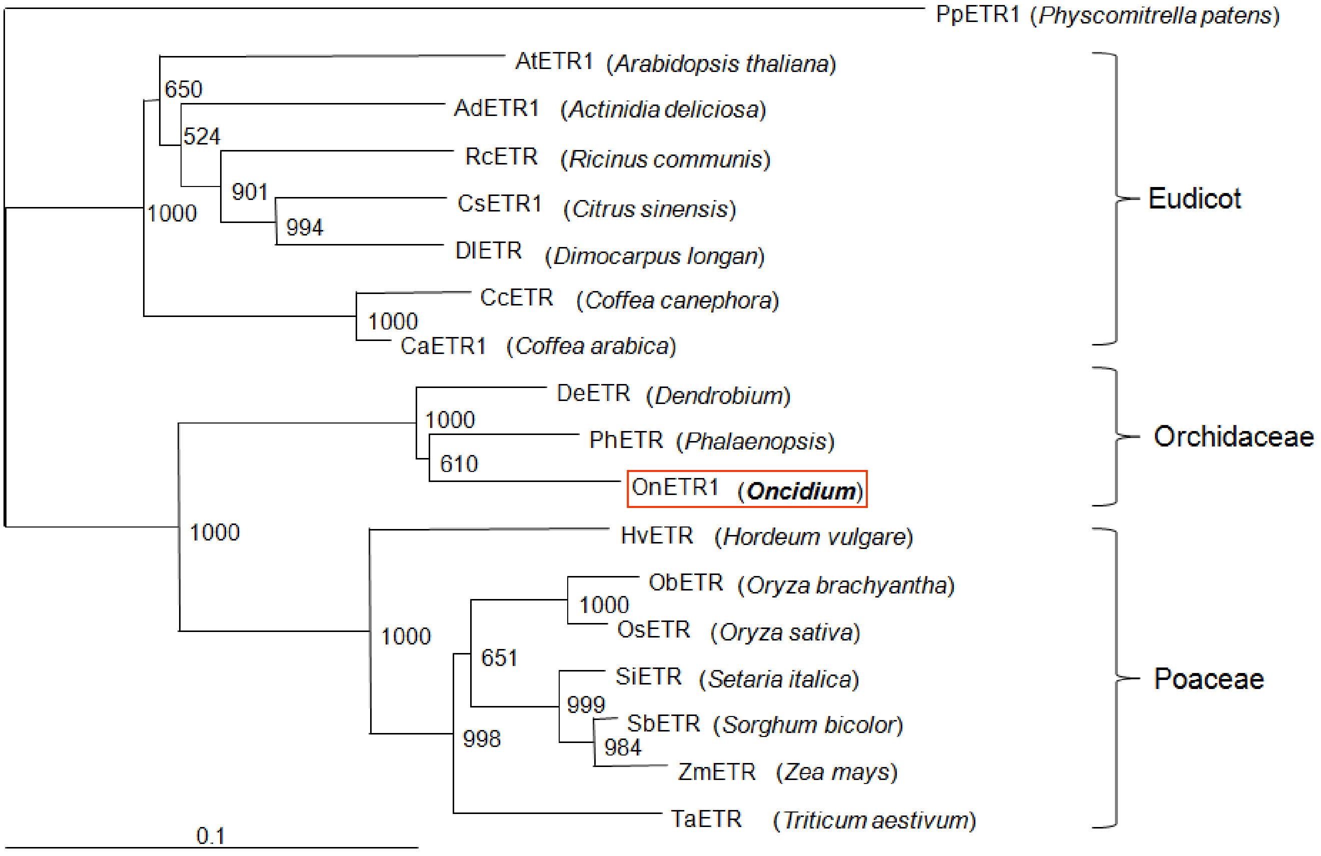

Supplementary Figure 5 Phylogenetic analysis of ETR1 proteins.
On the basis of amino acid sequence of the full-length protein, OnETR1 was closely related to orchid DeETR (*Dendrobium*) and PhETR (*Phalaenopsis*). Names of the plant species for each *ETR* gene are listed behind the protein names. Amino acid sequences of ETR proteins were obtained via the National Center for Biotechnology Information server (http://www.ncbi.nlm.nih.gov/). The multiple sequence alignment was performed by using ClustalW of DNA Data Bank of Japan (http://clustalw.ddbj.nig.ac.jp/top-e.html). The phylogenetic graph was generated by using the TREEVIEW program. Numbers on major branches indicate bootstrap percentages for 1,000 replicate analyses.

Supplementary Table 1. Oligo primer sequences used for gene cloning

Gene	Primer ID	Sequence	Restriction site	Used for
OnACS12	35Xba1-F	5'-CTCTAGAGTTTTCCTCCAAAAGGCAC-3'	XbaI	cDNA
	35Kpn1-R	5'-GCGGTACCGCAGATTCTCATTTCACTGAC-3'	KpnI	
OnETR1	OnETR1-F-BamHI	5'-GGATCCATGGAAGGCTGTGATTGCATT-3'	BamHI	cDNA
	OnETR1-R-KpnI	5'-GGTACCCTACAAACTTCTCTGATACCG-3'	KpnI	cDNA
	OnETR1(C65Y)-F	5'-TCCTTTACGGAGCAACCCA-3'		point mutation
	OnETR1(C65Y)-R	5'-GCTCCGTAAAGGACAATGAAG-3'		point mutation

Supplementary Table 2. Primer used for real-time quantitative PCR

Gene	Primer ID	Sequence
OnETR1	RT-OnETR1-F	5'-TGTCATTGTTTGTGTCTCTTGCTC-3'
	RT-OnETR1-R	5'-CTTAAATAGAAATGCCCGTCAGTAGG-3'
OnACS12	RT-ACS12-F	5'-TCTTAATATAGCCAAGGTCAATATCACAC-3'
	RT-ACS12-R	5'-CTAAGTCGCTCCATAACCACAGG-3'
CPS	RT-CPS-F	5'- CTATATGGAGATGAAGGAGAAGGAGAG -3'
	RT-CPS-R	5'- AGTGTGGGTGAAGAAGTTAGTTAGG -3'
KS	RT-KS-F	5'- GATAGGTGGATTGTGGAGAATAGATTG -3'
	RT-KS-R	5'- ATACGAGCATCAGATAGTTCAGGAG -3'
GA3ox1	RT-GA3ox1-F	5'- GCGTGCCTTTGGGACTTCTC -3'
	RT-GA3ox1-R	5'- CTTGCGTTGGACAGGTAGCC -3'
GA2ox1	RT-GA2ox1-F	5'- CAAGAGCGTGAGGCATAGGG-3'
	RT-GA2ox1-R	5'- AGTCAATGAAGGTCCAGCGAAG -3'
MYC2	RT-MYC2-F	5'- GAAGAAGATAAAGCAAACCCGAGAC-3'
	RT-MYC2-R	5'- CTCCTCATCAACAGCGTCATCC-3'
WRKY33	RT-WRKY33-F	5'-GCTGCTATTGCTGGTCACTCC -3'
	RT-WRKY33-R	5'-GAAGGTTGCTGTTGTTGTTATTGTTG -3'
GAI	RT-GAI-F	5'-GATGAATGAAGAAGACGACGGTAAC-3'
	RT-GAI-R	5'-TCGGTGAGCATAGAATCAAGCC-3'
RGL1	RT-RGL1-F	5'-GTTGGTTGGAAACTTGGTCAGC-3'
	RT-RGL1-R	5'-CGGTCGGATTGATTTGATTGTCG-3'
RGA	RT-RGA-F	5'-TTTAGGTTACAAAGTTAGGTCATCGG-3'
	RT-RGA-R	5'-CGGAAGAGGAGGAGGATTAAGC-3'
RGL2	RT-RGL2-F	5'-CAATGGCGGATAAGAAGAAGGC-3'
	RT-RGL2-R	5'-TAATGAACAGAGTCGTTTAAGACAGTAG-3'
UBQ10	RT-UBQ10-F	5'-CTCAGGCTCCGTGGTGGTATG-3'
	RT-UBQ	5'-GTGATAGTTTTCCCAGTCAACGTC-3'
FT	RT-AtFT-1	5'-ATCCGTTTAATAGATCAATCACTC-3'
	RT-AtFT-2	5'-TCACCAACCAATGGAGATATTC-3'
SOC1	RT-SOC1-F	5'-CTTCTAAACGTAAACTCTTGGGAGAAG-3'
	RT-SOC1-R	5'-CCTCGATTGAGCATGTTCCTATG-3'
CO	RT-CO-F	5'-GGCTCCTCAGGGACTCAC-3'
	RT-CO-R	5'-TGGGCGTTCTTGGGTGTG-3'
LFY	LEAFY-qPCR-F	5'-CACGCTCTTGATGCTCTCTCC-3'
	LEAFY-qPCR-R	5'-TGCTCCCTCTGTCTCTCTGTC-3'
MYB26	RT-MYB26-F	5'-ACAGATGGGCTCAAATTGCTAAAC-3'
	RT-MYB26-R	5'-TGATGATGATGACCGGATGATGG-3'
NST1	RT-NST1-F	5'-GCTTAACGGACCCACATCATATTC-3'
	RT-NST1-R	5'-TACGGAGATCGGACGGAAGG-3'
NST2	RT-NST2-F	5'-CGCCGTCGTTCAATGAGGAG-3'
	RT-NST2-R	5'-TCGTGATGGTGGTGTTGTTATGG-3'
MYB85	RT-MYB85-F	5'-GAGTGATAGTAACTTTGGTGTTGGTG-3'
	RT-MYB85-R	5'-ACATCTTCTCGTCATTGATTCTTTCC-3'
EDF1	EDF1-RT-1	5'-TCACAAACACAACAAATATGGAATACAG-3'
	EDF1-RT-2	5'-GCTTTGGAGTAGTAGAGATGGAGAG-3'
EDF2	EDF2-RT-1	5'-CTAAACAACACGCCGAGAAACAC-3'
	EDF2-RT-2	5'-GCCGAACCACCACCTGAACC-3'
EDF3	EDF3-RT-1	5'-ACACATATACATTCACATTACTAATCTC-3'
	EDF3-RT-2	5'-AATGGAATCTGTAGTTGTAGAGC-3'
EDF4	EDF4-RT-1	5'-CCGAAACATCACGCAGAGAAAC-3'
	EDF4-RT-2	5'-AACCCGACCCGCATCTAAATC-3'
ERF1	ERF1-RT-1	5'-GAGCAGTCCACGCAACAAAC-3‘
	ERF1-RT-2	5'-TCTCCGAAAGCGACTCTTGAAC-3‘
OnUBQ	OnUBQ-RT-1	5'-CCACGAAGGCGGAGAACAAG-3‘
	OnUBQ-RT-2	5'-AAGGCGAAGATTCAGGATAAGGAG-3‘

Supplementary Table 3. Flowering time and leaf numbers of wild-type and 35S::*OnACS12* plants that were grown under long-day (LD, 16 h light/ 8 h dark) conditions.

Genotype	No. of plants	Days of flowering[a]	Rosette leaves[b]
**WT**	**13**	**31.3 ± 1.1**	**8.9 ± 1.3**
**35S::*OnACS12***	**26**	**39.3 ± 2.5**	**9.3 ± 1.4**

[a] The average number of days for plants to develop an elongated inflorescence in two centimeters.

[b] The average number of rosette leaves for plants at the time of inflorescence in two centimeters.
